# Characterization of vB_Kpn_F48, a Newly Discovered Lytic Bacteriophage for *Klebsiella pneumoniae* of Sequence Type 101

**DOI:** 10.3390/v10090482

**Published:** 2018-09-09

**Authors:** Nagaia Ciacci, Marco Maria D’Andrea, Pasquale Marmo, Elisa Demattè, Francesco Amisano, Vincenzo Di Pilato, Maurizio Fraziano, Pietro Lupetti, Gian Maria Rossolini, Maria Cristina Thaller

**Affiliations:** 1 Department of Biology, University of Rome “Tor Vergata”, 00133 Rome, Italy; nagaia_ciacci@libero.it (N.C.); marco.dandrea@unisi.it (M.M.D.); pasquale.marmo@uniroma2.it (P.M.); fraziano@bio.uniroma2.it (M.F.); 2 Department of Medical Biotechnologies, University of Siena, 53100 Siena, Italy; 3 Center for Integrative Biology, CIBIO, University of Trento, 38122 Trento, Italy; elisa.dematte@unitn.it; 4 InBioS—Center for Protein Engineering, Department of Life Sciences, University of Liège, Sart-Tilman, 4000 Liège, Belgium; francesco.amisano@doct.uliege.be; 5 Department of Experimental and Clinical Medicine, University of Florence, 50134 Florence, Italy; vincenzo.dipilato@unifi.it (V.D.P.); gianmaria.rossolini@unifi.it (G.M.R.); 6 Department of Life Sciences, University of Siena, 53100 Siena, Italy; pietro.lupetti@unisi.it; 7 Clinical Microbiology and Virology Unit, Florence Careggi University Hospital, 50134 Florence, Italy

**Keywords:** bacteriophage, *Tevenvirinae*, *Klebsiella pneumoniae* carbapenemase (KPC), *K. pneumoniae*, sequence type 101, *Klebsiella pneumoniae* ST101

## Abstract

Resistance to carbapenems in *Enterobacteriaceae*, including *Klebsiella pneumoniae*, represents a major clinical problem given the lack of effective alternative antibiotics. Bacteriophages could provide a valuable tool to control the dissemination of antibiotic resistant isolates, for the decolonization of colonized individuals and for treatment purposes. In this work, we have characterized a lytic bacteriophage, named vB_Kpn_F48, specific for *K. pneumoniae* isolates belonging to clonal group 101. Phage vB_Kpn_F48 was classified as a member of *Myoviridae*, order *Caudovirales*, on the basis of transmission electron microscopy analysis. Physiological characterization demonstrated that vB_Kpn_F48 showed a narrow host range, a short latent period, a low burst size and it is highly stable to both temperature and pH variations. High throughput sequencing and bioinformatics analysis revealed that the phage is characterized by a 171 Kb dsDNA genome that lacks genes undesirable for a therapeutic perspective such integrases, antibiotic resistance genes and toxin encoding genes. Phylogenetic analysis suggests that vB_Kpn_F48 is a T4-like bacteriophage which belongs to a novel genus within the *Tevenvirinae* subfamily, which we tentatively named “*F48virus*”. Considering the narrow host range, the genomic features and overall physiological parameters phage vB_Kpn_F48 could be a promising candidate to be used alone or in cocktails for phage therapy applications.

## 1. Introduction

The extensive usage of antibiotics has led to the emergence and dissemination of antibiotic resistant bacteria, with an increasing number of reports in hospital settings of strains showing multidrug resistant (MDR), extremely drug resistant (XDR), or even pandrug resistant (PDR) phenotypes [1]. This situation is further complicated by the slow rate of discovery and development of new antibiotics, with the net result of a progressive reduction of effective therapeutic options for the management of serious infections [2,3]. Consequently, antibiotic resistance has become one of the major public health problems [3,4].

*Klebsiella pneumoniae* has emerged as one of the most challenging antibiotic-resistant pathogens, since it can cause a variety of infections (e.g., urinary tract infections, pneumonia, intra-abdominal infections, surgical site infections, bacteremia) and exhibits a remarkable propensity to acquire MDR and XDR phenotypes. Indeed, some clonal lineages of *K. pneumoniae* have acquired resistance determinants to most antibiotics available for treating these infections, including fluoroquinolones, aminoglycosides, expanded-spectrum cephalosporins and carbapenems, while retaining a notable propensity to disseminate in healthcare settings. Typical representatives of these “high-risk clones” (HiRiCs) include the Clonal Group (CG) 258 strains, which have played a major role in the dissemination of KPC-type carbapenemases, the CG15 strains, which are the second most prevalent CG responsible for ≈20% of all outbreaks, and the CG17, CG37, CG101 and CG147 strains [5].

In particular members of CG101, represented by strains of Sequence Type (ST) 101 and related variants (e.g., ST1789 and ST1633), are wide-spreading in several European and North African countries [6,7,8,9,10,11,12,13,14,15,16,17], and differently from other STs, exhibit a notable promiscuity in term of associated clinically-relevant resistance determinants, such as carbapenemases of the KPC, OXA-48, VIM and NDM types [6,11,18,19].

Among potential alternatives to conventional antibiotics against resistant pathogens, bacteriophages are being reconsidered with interest as valuable tools, given their ability to rapidly and selectively kill specific bacterial clones [20,21].

Here we describe the isolation and characterization of a novel bacteriophage of the *Myoviridae* family with a lytic activity specific to *K. pneumoniae* strains belonging to ST101 and other members of CG101.

## 2. Materials and Methods

### 2.1. Bacterial Host and Culture Conditions

The *K. pneumoniae* clinical strain 12C47, isolated in 2011 in Italy, was used as host for the isolation and propagation of the vB_Kpn_F48 bacteriophage. The strain was part of the collection characterized during the first national survey on carbapenem-resistant *Enterobacteriaceae* carried out in Italy in 2011, produced the KPC-2 carbapenemase, displayed a MDR phenotype, and was typed as ST101 by multi-locus sequence typing (MLST) [22].

### 2.2. Phage Isolation and Purification

Bacteriophage vB_Kpn_F48 was isolated from sewage wastewaters collected at the Florence Careggi University hospital by using the double-layer overlay technique and *K. pneumoniae* 12C47 as the indicator strain, as previously described [23]. Pure bacteriophage suspensions were obtained by three rounds of single plaque purification and re-infection of exponentially growing 12C47, as reported elsewhere [24]. Phage titer, expressed in plaque forming units (PFU)/mL, was estimated by the soft-agar overlay method [23].

### 2.3. Transmission Electron Microscopy (TEM)

Aliquots of bacteriophages particles preparations were centrifuged at 25,000× *g* for 1 h and suspended at ~10^12^ PFU/mL in SM buffer (10 mM Tris–HCl, pH 7.5; 100 mM NaCl; 10 mM MgSO_4_). Samples were processed by negative stain with 2% uranyl acetate and examined by a FEI Tecnai 12 (FEI, Eindhoven, The Netherlands) transmission electron microscope (TEM) fitted with an Osis Morada 2 × 4K CCD camera (Olympus, Shinjuku, Tokyo, Japan).

### 2.4. Determination of Bacteriophage Host Range

Host range of phage vB_Kpn_F48 was determined by the spot test technique using 61 previously characterized *K. pneumoniae* strains (Table 1), 24 enterobacterial isolates (*Klebsiella variicola*, *n* = 4; *Klebsiella quasipneumoniae* subsp. *quasipneumoniae*, *n* = 2; *Klebsiella oxytoca*, *n* = 1; *Enterobacter ludwigii*, *n* = 1; *Enterobacter gergoviae*, *n* = 1; *Enterobacter sakazakii*, *n* = 1; *Serratia marcescens*, *n* = 3; *Citrobacter freundii*, *n* = 1; *Providencia stuartii*, *n* = 1; *Edwardsiella tarda*, *n* = 1 and *Escherichia coli*, *n* = 8), 18 gram negative non fermenters (*Pseudomonas aeruginosa*, *n* = 14; *Acinetobacter baumannii*, *n* = 4) and 16 *E. coli* isolates obtained from stools of healthy children, as described by Hsu [25]. All strains with the exception of commensal *E. coli* were of nosocomial origin, and *K. pneumoniae* strains were representatives of clinically relevant and globally diffused clones. Each strain was grown in 5 mL of LB at 37 °C with shaking to an OD_600_ of 0.5, then centrifuged at 4500× *g* for 10 min and suspended in 5 mL of SM buffer. A 0.1 mL volume of the bacterial suspension was mixed with 4.5 mL of molten soft-agar, poured onto an LB agar (LBA) plate and, after solidification of the agar, 0.01 mL of phage suspension (titer ~10^7^ PFU/mL) were spotted on the overlay. Sensitivity to the vB_Kpn_F48 infection was tentatively assessed by the observation of a lysis halo at the spot after overnight (ON) incubation at 35 ± 2 °C, and then confirmed by the efficiency of plating (EOP) method as described below.

### 2.5. Preparation of High Titer Bacteriophage Suspensions

A large-scale preparation of vB_Kpn_F48 was obtained by the double-layer overlay technique. Briefly, an ON culture of strain 12C47 was centrifuged at 4500× *g* for 10 min and the bacterial pellet was suspended in 1/10 of the starting volume in SM buffer. A 0.1 mL aliquot of bacteriophage preparation with a titer of 1 × 10^6^ PFU/mL, previously filtered through 0.22 μm filter, was added to 0.2 mL of the bacterial suspension. After incubation of 20 min at 37 °C, the mixture was added to 4.5 mL of molten soft-agar and plated onto an LBA plate. Following an ON incubation at 37 °C, phages were recovered by adding 5 mL of SM buffer to each plate and incubating for 2 h at room temperature (RT) with gentle shaking. The soft-agar layer and SM buffer were then scraped and centrifuged at 4500× *g* for 10 min. The supernatant was collected, filtered through a 0.22 μm filter and stored at 4 °C. For long-term storage, phage aliquots were maintained in SM buffer supplemented with 25% glycerol at −80 °C.

### 2.6. Efficiency of Plating (EOP)

All *K. pneumoniae* isolates sensitive to phage vB_Kpn_F48 in the spot test assay (*n* = 14) were selected for the determination of EOP as described by Khan Mirzaei and Nilsson [27]. The EOP was computed as the ratio between PFU/mL on a sensitive strain and PFU/mL on the indicator strain and then used to rank each strain as “high productive” (EOP ≥ 0.5), “medium productive” (0.1 ≤ EOP < 0.5), “low productive” (0.001 < EOP < 0.1) or “inefficient” (EOP ≤ 0001). The assay was performed in triplicate for each combination of bacterial strain/phage dilution and results are reported as the mean of three observations.

### 2.7. One-Step Growth Curve

The dynamic change in phage particles number during a replicative cycle was monitored to determine the latency period, the eclipse period and the burst size of vB_Kpn_F48 as previously described [28] with minor modifications. Briefly, the host strain 12C47 was grown in aerobic conditions at 37 °C to mid-exponential phase (OD_600_ = 0.3–0.4, corresponding to ≈1–2 × 10^8^ CFU/mL). The culture was then centrifuged at 4000× *g* for 10 min at 4 °C, and suspended in a 1/10 volume of SM buffer; a 0.1 mL of bacteriophage was then added to 0.9 mL of the bacterial cell suspension to achieve a Multiplicity of Infection (MOI) of 0.01. Phages were allowed to absorb for 5 min at 37 °C in a water-bath, and then the mixture was centrifuged twice at 13,000× *g* for 2 min in order to eliminate the non-absorbed phages. The pellet was then suspended in 1 mL of SM buffer, diluted 1 × 10^−3^ in 10 mL of LB medium and incubated at 37 °C in a water-bath. Two aliquots were taken at 0, 5, 10, 20, 40, 50, 60, 70 and 80 min post-infection. One aliquot was diluted immediately and plated for phage titration, while the other was treated with 2% chloroform, shaken briefly, set aside for 10 min at RT and centrifuged. The aqueous phase of the latter aliquot was then titrated to determine intracellular phage concentrations. Results are reported as the average number of phages released per infected host cell, and the burst size was computed as the ratio of the final count of released phage particles to the initial count of infected bacterial cells during the latent period [29]. Experiments were performed in triplicate and results are reported as the mean of three observations ± SD.

### 2.8. Influence of Physical Agents on Phage Viability

Stability of vB_Kpn_F48 to different pH values was evaluated by suspending phages at approximately 10^7^ PFU/mL in aliquots of 1 mL SM buffer, previously adjusted with 1 M NaOH or 1 M HCl, to yield a pH range from 2.0 to 11.0 with intervals of 1 unit. Phage preparations were incubated at RT for 60 min. Stability of vB_Kpn_F48 to different temperatures was determined by incubation of phage preparations (≈10^7^ PFU/mL) at 25 °C (control), 40 °C, 50 °C, 60 °C, 65 °C and 70 °C for 10, 20, 40 and 60 min. In both cases, serial dilutions of each sample were tested against strain 12C47 in a double-layer agar assay to check for the lytic activity of the phage. Assays were performed in triplicate and the results are reported as the mean of phage counts (PFU/mL) ± SD.

### 2.9. Time-Kill Assay

To determine the activity of phage vB_Kpn_F48 against strain 12C47 in vitro, a time-kill assay was performed using a modified protocol [30]. The indicator strain was grown in 45 mL LB medium at 37 °C to OD_600_ = 0.2 (approximately 0.6 × 10^8^ CFU/mL) and infected with 5 mL phage suspension at about 5 × 10^8^ PFU/mL (MOI = 1), 5 × 10^7^ PFU/mL (MOI = 0.1) or 5 × 10^6^ PFU/mL (MOI = 0.01). A non-infected culture of 12C47 was set up as negative control. All samples were incubated at 37 °C with shaking, and 1 mL aliquots were collected at 30 min time intervals for 7 h and at 24 h post-infection. Each aliquot was centrifuged twice at 13,000× *g* for 2 min in order to eliminate the nonabsorbed phages and the pellet was then suspended in 1 mL of 0.9% NaCl. Each sample was finally serially diluted [31] and plated on LBA for enumeration of viable colonies following ON incubation at 35 ± 2 °C. Experiments were performed in triplicate and results are reported as the mean of three observations ± SD and expressed as log-transformed values (log CFU/mL) over time.

### 2.10. Rate of Appearance of Phage-Resistant Mutants

The rate of phage-resistant mutant emergence was determined by the method described by Merabishvili [32] by mixing 1 mL of exponentially growing (~10^8^ CFU/mL) culture of 12C47 with phage lysate to reach a MOI of 100. After 10 min of incubation at 37 °C with shaking, four 10-fold serial dilutions (10^1^–10^4^) in SM were prepared and 0.1 mL of each dilution was spread on LBA plates. Colonies were enumerated after overnight incubation at 35 °C. A non-infected culture of 12C47 was set up as negative control. The obtained colonies were isolated twice to assure phage-free bacterial cultures, and then tested against vB_Kpn_F48 by spot test to confirm their resistance to infection.

### 2.11. Extraction of Bacteriophage vB_Kpn_F48 DNA

Bacteriophage DNA was extracted from phage lysate with the kit Wizard^®^ DNA Clean-Up System (Promega, Madison, WI, USA) according to the method described by Gill [33]. Prior to DNA extraction, DNase (10 µg/mL) and RNase (10 µg/mL) were added to the phage lysate and the sample was incubated at RT for 2 h. After incubation, 2 volumes of precipitating solution (10% PEG-800, 1 M NaCl) were added, the mixture was incubated on ice for 1 h, centrifuged at 10,000× *g* for 10 min at 4 °C and the pellet was re-suspended in 0.5 mL of SM buffer. The obtained pellet was transferred to a new microcentrifuge tube and centrifuged for 5–10 s to remove any suspended particles. The supernatant was then transferred to a new tube and used for phage DNA extraction by using the above-mentioned kit. At the end of the extraction process, DNA samples were dissolved in 0.1 mL of sterile ddH_2_O preheated to 80 °C and stored at 4 °C.

### 2.12. Genome Sequencing

The genome of vB_Kpn_F48 was sequenced with the MiSeq instrument (Illumina Inc., San Diego, CA, USA) and a paired-ends approach (2 × 250 bp) by using the kit Illumina Nextera XT™. Direct Sanger sequencing was performed to determine the 5′- and 3′-ends of the phage genome and to close gaps occurring between contigs using the primers reported in Table S1.

### 2.13. Bioinformatics Analysis of Phage Genome

Reads obtained from genome sequencing were assembled using the SPAdes software [34] into 149 nodes. Nodes having a coverage <10× were considered of chromosomal origin or artifacts and thus removed from the subsequent analysis. A total of 10 nodes were then analyzed by BLASTN software using the nr database (http://blast.ncbi.nlm.nih.gov/) to remove additional nodes of chromosomal origin. A total of 8/10 nodes were then retained and ordered by using the characterized phage Bp7 (Accession number: NC_019500), the closest homolog deposited in the International Nucleotide Standard Database Collaboration (INSDC) databases, as template. A PCR approach coupled with Sanger sequencing was employed to verify the order of the nodes and to close gaps. Finally, direct Sanger sequencing on whole phage DNA preparations was employed to verify the 5′- and 3′-ends of the phage genome. A single DNA molecule of 170,764 bp was thus obtained and annotated by using the Rapid Annotation using Subsystem Technologies (RAST) web-service [35]. The on-line instance of tRNAScan-SE [36] was used to identify phage tRNA genes, while host tRNA genes were predicted using ARAGORN [37]. Automatic annotation was manually reviewed by BLASTP analysis against RefSeq proteins deposited in the INSDC databases. Analysis of conserved protein domains was performed by using the CD-Search tool [38] and the results were filtered to remove non-specific hits. Phylogenetic analysis were performed by using the large subunit terminases or Gp23 protein sequences of bacteriophages of the *Tevenvirinae* subfamily reported by the International Committee on Taxonomy of Viruses (ICTV) classification (https://talk.ictvonline.org/taxonomy/; last accessed 19 April 2018). In both cases closest homologues, i.e., proteins having a ≥85% identity in a BLAST search (https://blast.ncbi.nlm.nih.gov/), have been also included. Protein alignments and trees, generated by using the Neighbor Joining method and 1000 bootstraps, were obtained using ClustalW [39]. Analysis of phage genomes were conducted using the Genome-BLAST Distance Phylogeny (GBDP) method implemented by the VICTOR webserver, with settings recommended for prokaryotic viruses [40]. Phage lifestyles were predicted using Phage Classification Tool Set (PHACTS) [41].

## 3. Results

### 3.1. Phage Isolation and Morphological Characterization

**Figure 1:**
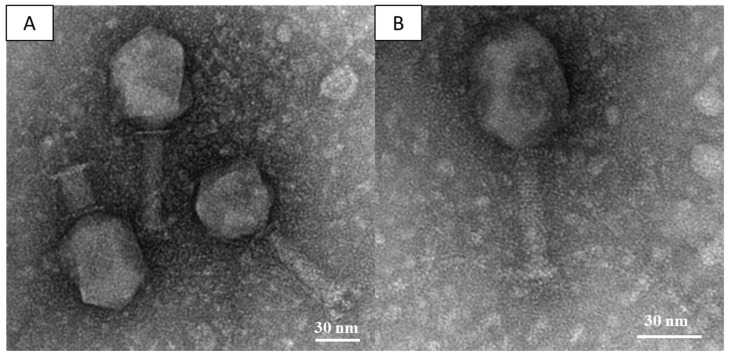
(**A**,**B**) Electron micrograph of phage vB_Kpn_F48 negatively stained with uranyl acetate. Bars indicate 30 nm.

The strain 12C47, an ST101 KPC-producing *Klebsiella pneumoniae* (KPC-Kp) isolated in Italy in 2011, was used as host to investigate the presence of virulent phages in a wastewater sample of a large tertiary-care teaching hospital located in central Italy, where KPC-Kp have been endemic since 2009 [22,42]. Results from this experiment revealed the presence of ≈50 clear small plaques (diameter < 1 mm) all characterized by a similar morphology, suggesting the presence of a single lytic phage. One plaque was picked up, and used for the propagation of the selected phage, named vB_Kpn_F48. Results from TEM analysis showed that vB_Kpn_F48 was characterized by a prolated head, approximately 120 nm × 80 nm, which is connected by an apparent collar to a helical, contractile tail (≈100 nm long) that ends with a baseplate provided with tail pins and several tail fibers (Figure 1). These morphological features suggested that vB_Kpn_F48 belongs to the family *Myoviridae*, order *Caudovirales*.

### 3.2. Phage Host Range

A total of 103 clinical isolates (61 *K. pneumoniae* of different clonal lineages and 42 gram negatives not belonging to the *K. pneumoniae* species) plus 16 commensal *E. coli* from healthy children were used to evaluate the host range of vB_Kpn_F48 (Table 1). Results demonstrated that this phage had a lytic activity specific to *K. pneumoniae* strains of ST101 (*n* = 11) and single locus variants thereof (i.e., ST1633, *n* = 1; ST2502, *n* = 2). All the other tested strains, including *K. pneumoniae* (*n* = 47), belonging to different clonal lineages (i.e., CG258) [43,44], were found to be insensitive to the infection by vB_Kpn_F48.

### 3.3. EOP of Phage vB_Kpn_F48

The EOP analysis revealed a high productive infection of phage vB_Kpn_F48 for 9 of the 14 strains sensitive to vB_Kpn_F48 in the spot assay. A medium production has been observed for the isolate belonging to ST1633, while the remaining four strains resulted in inefficient production (Table 2). An inefficient production of the phage could be explained either by an abortive infection or a lysis from without phenomenon, two processes that involve the adsorption of a high number of phage particles onto the bacterial cell wall and end with the rapid disruption of the bacterial cell-forming clear zones on a bacterial plate without phage production [45].

### 3.4. Latency Period, Eclipse Period and Burst Size Determination

**Figure 2:**
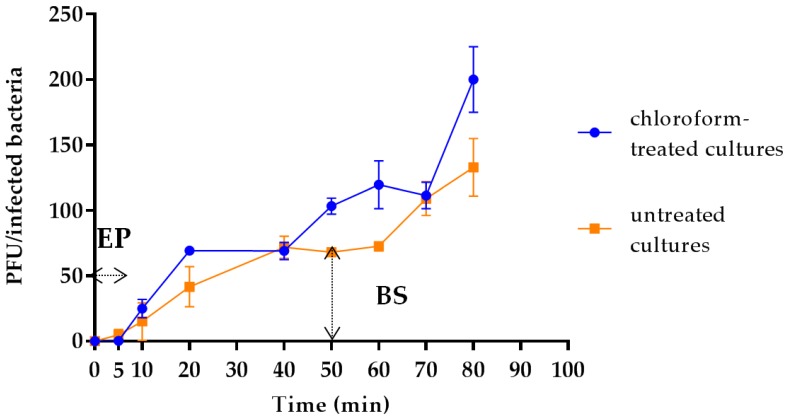
One-step growth curve of bacteriophage vB_Kpn_F48. The PFU per infected cell at different time points in chloroform-treated cultures (blue) and in untreated cultures (orange) are shown. EP: eclipse period; BS: burst size. Each data point is the mean from three experiments. Standard deviations are shown as vertical lines.

Results from one-step growth experiments showed a triphasic curve indicating that vB_Kpn_F48 was characterized by relatively short eclipse and latency periods (both less than 10 min) followed by a rise period of 40 min and a growth plateau reached in 40 min (Figure 2). The burst size of vB_Kpn_F48 was computed as 72 phages particles per infected bacteria. The increase in phage titer reported after one hour of infection represents the beginning of the second replication cycle, and thus data from this phase were not considered for the burst size computation.

### 3.5. Sensitivity to Physical Parameters

**Figure 3:**
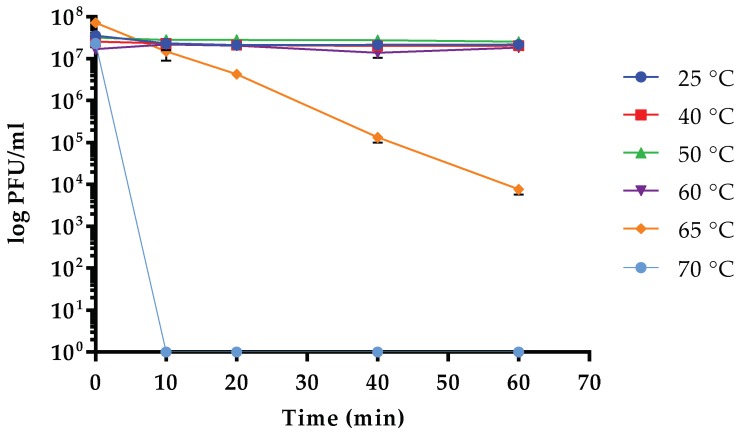
Thermal stability of vB_Kpn_F48. Each data point is the mean from three experiments. Standard deviations are shown as vertical lines.

**Figure 4:**
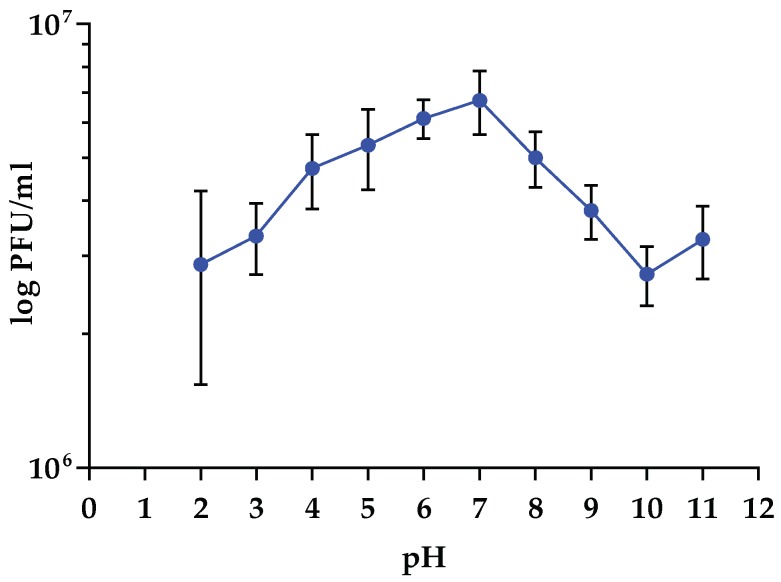
pH stability test of vB_Kpn_F48. Each data point is the mean from three experiments. Standard deviations are shown as vertical lines.

Data obtained from stability test vs. temperature variations demonstrated that the infectivity of vB_Kpn_F48 was overall stable in the range between 25 and 60 °C. Conversely, a high decrease of the infective capacity (~4 log) was observed after incubation at 65 °C for 60 min with a complete inactivation of vB_Kpn_F48 after incubation at 70 °C for 10 min (Figure 3). Results of stability test at different pH values indicated that vB_Kpn_F48 retained the maximum infectivity after incubation at pH values ranging from 4.0 to 8.0, while a reduction of approximately 40% was detected at pH 2.0 and pH 3.0. A decrease in phage infectivity was also detected at pH 9.0 (25%) and at pH 10.0 (45%). Anyway, a high phage viability was observed in all the tested conditions (Figure 4).

### 3.6. Killing Dynamic of vB_Kpn_F48 against the 12C47 Strain

**Figure 5:**
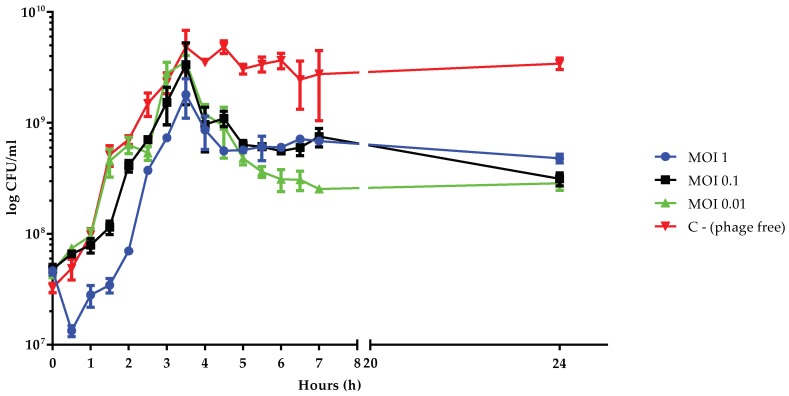
Time-kill assays with bacterial host in exponential phase. Viable cell count (log CFU/mL) of 12C47 cultures infected with vB_Kpn_F48 at MOI of 1 (blue), MOI of 0.1 (black), MOI of 0.01 (green) and the uninfected control (red) are shown. Each data point is the mean from three experiments. Standard deviations are shown as vertical lines.

To evaluate the killing effect of phage vB_Kpn_F48 against the 12C47 strain, liquid cultures of the bacterial host in the exponential phase were infected with the phage at three different MOI and cell number was monitored over time (Figure 5). Data obtained from this assay showed a sigmoid curve for all conditions characterized by a lag phase that is directly correlated with the MOI. In particular, lag phases of approximately 1, 2, or 2.5 h were observed by using MOIs of 0.01, 0.1 or 1, respectively. After 4 h the cultures, at all the tested MOIs, showed a 0.5–1 log reduction in cell count, even if the reduction of viable cells doesn’t correlate with MOIs, with MOI of 0.01 that resulted in the higher killing effect. A similar trend, i.e., the absence of an inverse correlation between MOI and bacterial growth, has been previously observed and correlated to the appearance of phage-resistant/persister bacteria following a random fluctuation model [47,48].

### 3.7. Frequency of Emergence of Phage-Resistant Mutants

The frequency of emergence of phage-resistant mutant bacterial cells was estimated to be 1.5 × 10^−7^ mutants per total cells count for bacteriophage vB_Kpn_F48. This frequency is similar to that obtained for other *Myoviruses*, e.g., phage Acibel004 infecting *A. baumannii* that showed a mutation frequency rate of 1.2 × 10^−7^ mutants per total cells count [32]. Resistant mutants were phenotypically undistinguishable from parental strain.

### 3.8. Genome Analysis

Molecular characterization, performed by high-throughput DNA sequencing and Sanger sequencing for the resolution of the 5′- and 3′-ends and gap closing, revealed that the genome of vB_Kpn_F48 is composed by a linear dsDNA molecule of 170,764 bp and a GC content of 40.8%. No repeated terminal sequences were detected at the 5′- and 3′-ends, consistent with other members of the *Myoviridae* family. A total of 283 putative coding regions (CDSs) have been detected by RAST analysis. Most of the predicted CDSs (*n* = 239, corresponding to 84.4%) are encoded on the same strand. The shortest CDS encoded a putative protein of 37 amino acid residues (CDS 140), while the longest encodes a putative protein of 1403 amino acid residues (CDS 156). The majority of predicted CDSs have an ATG initial codon (*n* = 261, corresponding to 92.2%), 17 start with GTG (6%), and the remaining five with TTG (1.8%). A specific putative function (transcriptional factors, structural proteins, enzymes involved in the replication and degradation of DNA) could be assigned to 120 of 283 deduced proteins (42.4%), with 21 of 120 (17.5%) showing high sequence identity to proteins described in phages of the *Tevenvirinae* subfamily. No specific function was assigned to the remaining 163 CDSs (57.6%; Table S2). No significant similarity with known antibiotic resistance determinants, virulence or toxin proteins, or with elements commonly associated with lysogeny (i.e., integrases, repressors and antirepressors) was revealed. A lytic lifestyle was predicted by PHACTS analysis, and this prediction was also supported by the presence of the *ndd* and *denB* genes whose deduced products shared a high degree of similarity (73% and 63% respectively) with the correspondent endonucleases encoded by the T4 phage. A lysozyme-like superfamily domain was detected in three deduced aminoacid sequences (AUO78644, AUO78846 and AUO78885) which were homologous to the T4 Gpe, Gp25 and Gp5 proteins (85%, 78% and 79% similarity, respectively). A total of 8 tRNA were detected by the tRNA-SCAN tool, and 7 were confirmed also by using ARAGORN. Genes encoding tRNAs are organized in a modular fashion, a feature common among the *Caudovirales*, and are equally distributed upstream and downstream the regions flanking ORF 274-ORF 278*.*

**Figure 6:**
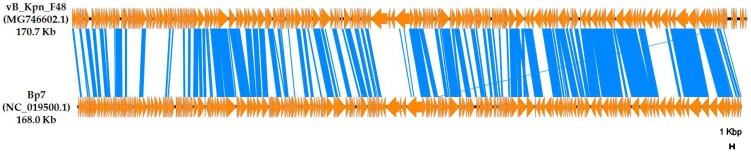
Comparative analysis between vB_Kpn_F48 and the Bp7 phage. Genetic map was constructed using EasyFig. ORFs are represented by orange arrows. Genome fragments having an identity ≥64% are connected by blue areas.

Comparative analysis of whole genome performed against phages deposited in the INSDC databases revealed that vB_Kpn_F48 is most closely related with the characterized *E. coli* Bp7 phage (overall nucleotide identity, 52.3%), a member of the *Myoviridae* family, *Tevenvirinae* subfamily [49,50] (Figure 6).

The nucleotide sequence of vB_Kpn_F48 was deposited in the GenBank database under accession number MG746602.

### 3.9. Phylogenetic Analysis

**Figure 7:**
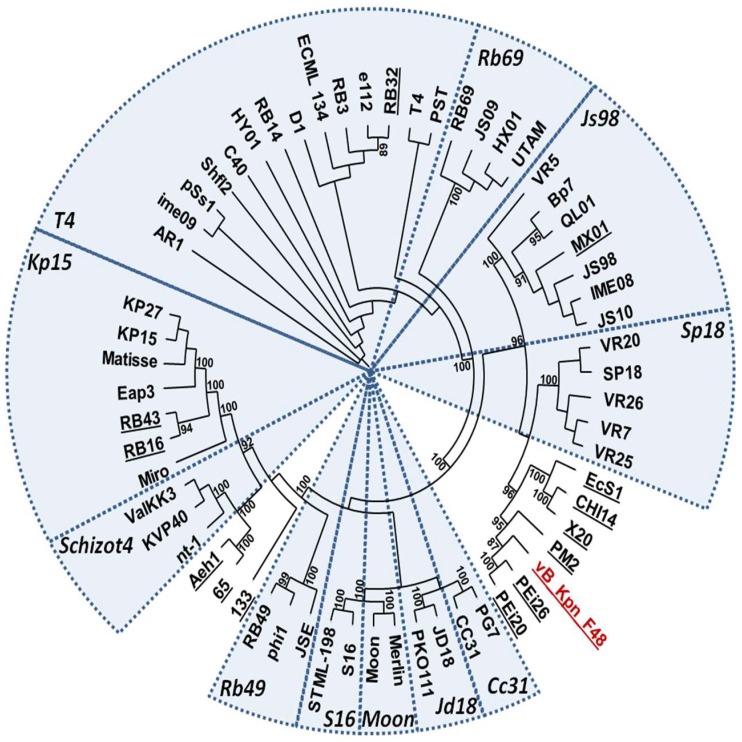
Phylogenetic tree of large subunit terminase of vB_Kpn_F48 (red) and homologues proteins of other members of the *Tevenvirinae* subfamily. Distinct genera are highlighted by circular sectors. Phages not assigned to specific genera by the ICTV classification (accessed 19 April 2018) are underlined. Bootstrap values ≥ 85% are indicated.

Results of BLASTP searches using the large terminase subunit revealed that vB_Kpn_F48 is a member of *Tevenvirinae* subfamily of *Myoviridae*. A phylogenetic tree of large terminase subunits gave results highly consistent with the current ICTV taxonomy, but suggested that vB_Kpn_F48 could not be assigned to any of the known *genus* (Figure 7). This observation was also confirmed by the analysis of Gp23 proteins. A phylogenetic analysis of the whole phage genome, performed by using the VICTOR web service, confirmed these findings, strongly suggesting that vB_Kpn_F48 is a member of a novel genus of the *Tevenvirinae* subfamily (Figure S1).

## 4. Discussion

The spread of antibiotic-resistant pathogens constitutes a serious matter in the clinical setting, given the scarcity of available treatment options. In particular, resistance to carbapenems and its association to MDR phenotypes in *Enterobacteriaceae*, including *K. pneumoniae*, is one of the major clinical challenges.

Among the most diffused HiRiCs of *K. pneumoniae*, those of CG101 are on the rise. Their propensity to acquire different clinically relevant resistance determinants, together with the ability to produce biofilm and several virulence factors are likely key factors for their success [51].

Bacteriophages could provide a valuable tool to control the dissemination of MDR microorganisms, especially HiRiCs. In addition, bacteriophages could be used for decolonization purposes, leading to the reduction of the load of specific bacterial clones and thus limiting the chance of their spreading. Due to their high specificity, phages can avoid also the alteration of the natural microbiological equilibrium occurring in the human microbiota when conventional antibiotics are used [52]. These features, together with their capacity to overcome bacterial antibiotic-resistance, make bacteriophages potential alternatives for clinical applications in infections caused by MDR bacteria [53,54].

In this work, we have described a novel lytic bacteriophage characterized by a narrow spectrum of lytic activity towards isolates of CG101. It is worth noting that *K. pneumoniae* isolates belonging to ST101 and variants thereof are spreading not only in Europe, including Italy, but also in other countries such as Algeria and Malaysia, where hospital outbreaks sustained by *K. pneumoniae* of ST101 have been reported [12,13]. To the best of our knowledge, this work represents the first report of a lytic phage that specifically targets strains of CG101. It is interesting to notice by the EOP test that both strains of ST2502 (CG101) gave an inefficient production of vB_Kpn_F48, maybe due to a reduced efficiency of phage recognition based on genetic mutations in this clonal lineage. Furthermore, all strains of CG101 used in this work were predicted from *wzi* sequencing to express a K17 serotype [26]. This may suggest that, differently from *E. coli* phage T4 that targets lipopolysaccharide or outer membrane OmpC as receptors [55], the cell structures recognized by vB_Kpn_F48 are components of the capsular polysaccharide. The lack of strains of the K17 serotype not belonging to CG101 in our collection didn’t allow to test such a correlation, but genomic analyses of phage resistant mutants are currently ongoing and will be the subject of future investigations aimed to characterize vB_Kpn_F48 receptors.

Morphological characterization performed by TEM showed that vB_Kpn_F48 is a member of the family *Myoviridae*, order *Caudovirales*. Results of phylogenetic analysis performed using markers previously employed in several phylogenetic studies of the T4- and T7-like phages [56,57,58,59], strongly suggested that vB_Kpn_F48 is a member of a novel genus of the *Tevenvirinae* subfamily. This finding is also supported by the phylogenetic analysis of the whole phage genome which shows a full consistency of the obtained trees and current ICTV taxonomy, with very high bootstrap values, and again confirmed that vB_Kpn_F48 forms a distinct clade of the *Tevenvirinae* subfamily. For these reasons, we propose that vB_Kpn_F48 represents a new genus within the *Tevenvirinae*, which we tentatively named “*F48viruses*”.

Physiological characterization showed that vB_Kpn_F48 is characterized by relatively short latent and eclipse periods and a low burst size of 72 phage particles released per infected bacteria. The result is a slight reduction in bacterial cells count over time as highlighted by the killing-curve, with a low frequency of occurrence of phage-resistant mutant, one of the major important infection parameters for the therapeutic use of bacteriophages. Results from stability test to physicochemical parameters demonstrated that vB_Kpn_F48 is exceedingly stable to temperature variations if compared to other lytic phages of the *Myoviridae* family targeting *K. pneumoniae*, e.g., the phage vB_KpnM_KP15 [60] which showed a 100-fold decrease in titer after 10 min at 60 °C. Similar results were obtained for stability to pH variations. These features, together with the host specificity, its close genetic relatedness to a well-known group of strictly lytic phages, together with the absence of the genes associated with lysogeny and the relatively low frequency of occurrence of phage-resistant mutants, make vB_Kpn_F48 an excellent candidate of interest for potential clinical applications [61], such as decontamination, decolonization and/or therapy. Finally, the presence in vB_Kpn_F48 of several T4-like putative endonucleases (e.g., Ndd and DenB homologues) suggests that this phage is able to prevent the “superspreader” effect [62], i.e., the release of intact, transformable plasmid DNA that upon lysis can be transferred horizontally by transformation, making vB_Kpn_F48 a promising candidate to hinder the colonization process.

## Figures and Tables

**Table 1:** Host spectrum of vB_Kpn_F48 towards *K. pneumoniae* clinical isolates. For each combination of sequence type/capsular genotype, the number of tested strains, the deduced capsular type basing on the *wzi* sequencing method and the result of the lysis test are reported (- = all strains were not lysed; + = all strains were lysed; ND = Not deducible).

No. of Strains ^a^	Sequence Type	Capsular Genotype ^b^	Deduced K-Type	Lysis
11	ST101	*wzi*137	K17	+
2	ST2502	*wzi*137	K17	+
1	ST11	*wzi*75	ND	-
3	ST14	*wzi*2	K2	-
2	ST15	*wzi*24	K24	-
1	ST1633	*wzi*137	K17	+
1	ST208	*wzi*35	K35	-
3	ST258	*wzi*154	ND	-
1	ST258	*wzi*29	K41	-
1	ST1879	*wzi*154	ND	-
6	ST307	*wzi*173	ND	-
1	ST340	*wzi*50	K15/K17/K50/K51/K52	-
4	ST37	*wzi*96	K38	-
1	ST42	*wzi*41	ND	-
1	ST45	*wzi*101	ND	-
2	ST512	*wzi*154	ND	-
1	ST540	*wzi*33	ND	-
1	ST554	*wzi154*	ND	-
2	ST641	*wzi*38	K38	-
1	ST745	*wzi*154	ND	-
1	ST23	*wzi1*	K1	-
1	ST11	*wzi174*	ND	-
1	ST489	*wzi171*	ND	-
1	ST3	*wzi3*	K3	-
2	ST16	*wzi50*	K15/K17/K50/K51/K52	-
2	ST730	*wzi356*	ND	-
1	ST147	*wzi64*	K14/K64	-
1	ST475	*wzi201-like*	ND	-
1	ST25	*wzi72*	K2	-
1	ST54	*wzi14*	K14	-
1	ST859	*wzi2*	K2	-
1	ST66	*wzi4*	K2	-
1	ST340	*wzi19*	K19	-

^a^ tested strains were from different hospitals, samples, patients and countries (Italy and Czech Republic) and were mostly epidemiological unrelated. ^b^ capsular genotyping was performed using the *wzi* method described by Brissè and colleagues [26].

**Table 2:** Efficiency of plating (EOP) for phage vB_Kpn_F48 on *K. pneumoniae* isolates sensitive to the phage infection. For each isolate, the strain identifier, the sequence type and the EOP are reported. Each EOP value is the mean of three observations ± SD. EOP of the original strain of isolation is marked in bold and the type of phage production is ranked according to Viazis and colleagues [46].

ID	Sequence Type	EOP	Production
12C47	ST101	1	High
12C73	ST101	0.9 ± 0.2	High
KPC174	ST1633	0.44 ± 0.06	Medium
5559	ST101	0.5 ± 0.2	High
5583	ST2502	0 ^a^	Inefficient
C002	ST101	1.9 ± 0.7	High
K13	ST101	2.62 ± 0.01	High
K18	ST101	<0.001 ^b^	Inefficient
6071	ST2502	0	Inefficient
12C29	ST101	2.2 ± 0.7	High
5546	ST101	3.9 ± 0.3	High
12C72	ST101	3.1 ± 0.3	High
KPC220	ST101	0	Inefficient
494647	ST101	0.9 ± 0.2	High

^a^ EOP = 0 indicates susceptibility to phage infection observed in the spot test but not in the EOP assay. ^b^ EOP < 0.001 indicates that the PFU produced by the phage on the target bacterium was more than 1000 times less than those observed on the indicator strain.

## Supplementary Materials

The following are available online at http://www.mdpi.com/1999-4915/10/9/482/s1, Table S1: Primers used for the vB_Kpn_F48 genome characterization, Table S2: General features of the coding regions of phage vB_Kpn_F48, Figure S1: Phylogenomic GBDP tree inferred using the formula D4 yielding the maximum average support of 71% among the three different formulas obtained by the VICTOR web server.

## References

[1] Rossolini G.M., Arena F., Pecile P., Pollini S. (2014). Update on the antibiotic resistance crisis. Clin. Opin. Pharmacol..

[2] Bush K. (2010). Alarming β-lactamase-mediated resistance in multidrug-resistant *Enterobacteriaceae*. Curr. Opin. Microbiol..

[3] Michael C.A., Dominey-Howes D., Labbate M. (2014). The antimicrobial resistance crisis: Causes, consequences, and management. Front. Public Health.

[4] Prestinaci F., Pezzotti P., Pantosti A. (2015). Antimicrobial resistance: A global multifaceted phenomenon. Pathog. Glob. Health..

[5] Navon-Venezia S., Kondratyeva K., Carattoli A. (2017). *Klebsiella pneumoniae*: A major worldwide source and shuttle for antibiotic resistance. FEMS Microbiol. Rev..

[6] Mammina C., Bonura C., Aleo A., Fasciana T., Brunelli T., Pesavento G., Degl’Innocenti R., Nastasi A. (2012). Sequence type 101 (ST101) as the predominant carbapenem-non-susceptible *Klebsiella pneumoniae* clone in an acute general hospital in Italy. Int. J. Antimicrob. Agents..

[7] Bonura C., Giuffrè M., Aleo A., Fasciana T., Di Bernardo F., Stampone T., Giammanco A., Palma D.M., Mammina C., MDR-GN Working Group (2015). An update of the evolving epidemic of *bla*_KPC_ carrying *Klebsiella pneumoniae* in Sicily, Italy, 2014: Emergence of multiple non-ST258 clones. PLoS ONE.

[8] Conte V., Monaco M., Giani T., D’Ancona F., Moro M.L., Arena F., D’Andrea M.M., Rossolini G.M., Pantosti A., AR-ISS Study Group on Carbapenemase-Producing *K. pneumoniae* (2016). Molecular epidemiology of KPC-producing *Klebsiella pneumoniae* from invasive infections in Italy: Increasing diversity with predominance of the ST512 clade II sublineage. J. Antimicrob. Chemother..

[9] Mshana S.E., Fritzenwanker M., Falgenhauer L., Domann E., Hain T., Chakraborty T., Imirzalioglu C. (2015). Molecular epidemiology and characterization of an outbreak causing *Klebsiella pneumoniae* clone carrying chromosomally located *bla*_CTX-M-15_ at a German University-Hospital. BMC Microbiol..

[10] Skalova A., Chudejova K., Rotova V., Medvecky M., Studentova V., Chudackova E., Lavicka P., Bergerova T., Jakubu V., Zemlickova H. (2017). Molecular Characterization of OXA-48-Like-Producing *Enterobacteriaceae* in the Czech Republic and Evidence for Horizontal Transfer of pOXA-48-Like Plasmids. Antimicrob. Agents Chemother..

[11] Oteo J., Pérez-Vázquez M., Bautista V., Ortega A., Zamarrón P., Saez D., Fernández-Romero S., Lara N., Ramiro R., Aracil B. (2016). The spread of KPC-producing *Enterobacteriaceae* in Spain: WGS analysis of the emerging high-risk clones of *Klebsiella pneumoniae* ST11/KPC-2, ST101/KPC-2 and ST512/KPC-3. J. Antimicrob. Chemother..

[12] Loucif L., Kassah-Laouar A., Saidi M., Messala A., Chelaghma W., Rolain J.M. (2016). Outbreak of OXA-48-producing *Klebsiella pneumoniae* involving a Sequence Type 101 clone in Batna University Hospital, Algeria. Antimicrob. Agents Chemother..

[13] Low Y.M., Yap P.S., Abdul Jabar K., Ponnampalavanar S., Karunakaran R., Velayuthan R., Chong C.W., Abu Bakar S., Md Yusof M.Y., Teh C.S. (2017). The emergence of carbapenem resistant *Klebsiella pneumoniae* in Malaysia: Correlation between microbiological trends with host characteristics and clinical factors. Antimicrob. Resist. Infect. Control.

[14] De Laveleye M., Huang T.D., Bogaerts P., Berhin C., Bauraing C., Sacré P., Noel A., Glupczynski Y., multicenter study group (2017). Increasing incidence of carbapenemase-producing *Escherichia coli* and *Klebsiella pneumoniae* in Belgian hospitals. Eur. J. Clin. Microbiol. Infect. Dis..

[15] Del Franco M., Paone L., Novati R., Giacomazzi C.G., Bagattini M., Galotto C., Montanera P.G., Triassi M., Zarrilli R. (2015). Molecular epidemiology of carbapenem resistant *Enterobacteriaceae* in Valle d’Aosta region, Italy, shows the emergence of KPC-2 producing *Klebsiella pneumoniae* clonal complex 101 (ST101 and ST1789). BMC Microbiol..

[16] Poulou A., Voulgari E., Vrioni G., Koumaki V., Xidopoulos G., Chatzipantazi V., Markou F., Tsakris A. (2013). Outbreak caused by an ertapenem-resistant, CTX-M-15-producing *Klebsiella pneumoniae* sequence type 101 clone carrying an OmpK36 porin variant. J. Clin. Microbiol..

[17] Potron A., Poirel L., Rondinaud E., Nordmann P. (2013). Intercontinental spread of OXA-48 β-lactamase-producing *Enterobacteriaceae* over a 11-year period, 2001 to 2011. Eurosurveillance.

[18] Jayol A., Poirel L., Dortet L., Nordmann P. (2016). National survey of colistin resistance among carbapenemase-producing *Enterobacteriaceae* and outbreak caused by colistin-resistant OXA-48-producing *Klebsiella pneumoniae*, France, 2014. Eurosurveillance.

[19] Pitart C., Solé M., Roca I., Fàbrega A., Vila J., Marco F. (2011). First outbreak of a plasmid-mediated carbapenem-hydrolyzing OXA-48 beta-lactamase in *Klebsiella pneumoniae* in Spain. Antimicrob. Agents Chemother..

[20] Pires D.P., Cleto S., Sillankorva S., Azeredo J., Lu T.K. (2016). Genetically engineered phages: A review of advances over the last decade. Microbiol. Mol. Biol. Rev..

[21] Domingo-Calap P., Georgel P., Bahram S. (2016). Back to the future: Bacteriophages as promising therapeutic tools. HLA.

[22] Giani T., Pini B., Arena F., Conte V., Bracco S., Migliavacca R., Pantosti A., Pagani L., Luzzaro F., Rossolini G.M., AMCLI-CRE Survey Participants (2013). Epidemic diffusion of KPC carbapenemase-producing *Klebsiella pneumoniae* in Italy: Results of the first countrywide survey, 15 May to 30 June 2011. Eurosurveillance.

[23] Adams M. (1959). Bacteriophage.

[24] Di Lallo G., Evangelisti M., Mancuso F., Ferrante P., Marcelletti S., Tinari A., Superti F., Migliore L., D’Addabbo P., Frezza D. (2014). Isolation and partial characterization of bacteriophages infecting *Pseudomonas syringae* pv. *actinidiae*, causal agent of kiwifruit bacterial canker. J. Basic Microbiol..

[25] Hsu C.R., Lin T.L., Pan Y.J., Hsieh P.F., Wang J.T. (2013). Isolation of a bacteriophage specific for a new capsular type of *Klebsiella pneumoniae* and characterization of its polysaccharide depolymerase. PLoS ONE.

[26] Brisse S., Passet V., Haugaard A.B., Babosan A., Kassis-Chikhani N., Struve C., Decré D. (2013). *wzi* Gene sequencing, a rapid method for determination of capsular type for *Klebsiella* strains. J. Clin. Microbiol..

[27] Khan Mirzaei M., Nilsson A.S. (2015). Isolation of phages for phage therapy: A comparison of spot tests and ffficiency of plating analyses for determination of host range and efficacy. PLoS ONE.

[28] D’Andrea M.M., Marmo P., Henrici De Angelis L., Palmieri M., Ciacci N., Di Lallo G., Demattè E., Vannuccini E., Lupetti P., Rossolini G.M. (2017). φBO1E, a newly discovered lytic bacteriophage targeting carbapenemase-producing *Klebsiella pneumoniae* of the pandemic Clonal Group 258 clade II lineage. Sci. Rep..

[29] Adams M.H. (1959). Bacteriophage.

[30] Clinical and Laboratory Standards Institute (1999). Methods for Determining Bactericidal Activity of Antimicrobial Agents: Approved Guidelines.

[31] Murray P., Baron E., Jorgensen J., Pfaller M., Yolken R. (2003). Manual of Clinical Microbiology.

[32] Merabishvili M., Vandenheuvel D., Kropinski A.M., Mast J., de Vos D., Verbeken G., Noben J.P., Lavigne R., Vaneechoutte M., Pirnay J.P. (2014). Characterization of Newly Isolated Lytic Bacteriophages Active against *Acinetobacter baumannii*. PLoS ONE.

[33] Gill J.J. (2015). Phage Genomic DNA Extraction. https://openwetware.org/wiki/Gill:Phage_genomic_DNA_extraction.

[34] Bankevich A., Nurk S., Antipov D., Gurevich A.A., Dvorkin M., Kulikov A.S., Lesin V.M., Nikolenko S.I., Pham S., Prjibelski A.D. (2012). SPAdes: A new genome assembly algorithm and its applications to single-cell sequencing. J. Comput. Biol..

[35] Aziz R.K., Bartels D., Best A.A., DeJongh M., Disz T., Edwards R.A., Formsma K., Gerdes S., Glass E.M., Kubal M. (2008). The RAST Server: Rapid annotations using subsystems technology. BMC Genom..

[36] Lowe T.M., Chan P.P. (2016). tRNAscan-SE On-line: Integrating search and context for analysis of transfer RNA genes. Nucleic Acids Res..

[37] Laslett D., Canback B. (2004). ARAGORN, a program to detect tRNA genes and tmRNA genes in nucleotide sequences. Nucleic Acids Res..

[38] Marchler-Bauer A., Derbyshire M.K., Gonzales N.R., Lu S., Chitsaz F., Geer L.Y., Geer R.C., He J., Gwadz M., Hurwitz D.I. (2015). CDD: NCBI’s conserved domain database. Nucleic Acids Res..

[39] Larkin M.A., Blackshields G., Brown N.P., Chenna R., McGettigan P.A., McWilliam H., Valentin F., Wallace I.M., Wilm A., Lopez R. (2007). Clustal W and Clustal X version 2.0. Bioinformatics.

[40] Meier-Kolthoff J.P., Göker M. (2017). VICTOR: Genome-based phylogeny and classification of prokaryotic viruses. Bioinformatics.

[41] McNair K., Bailey B.A., Edwards R.A. (2012). PHACTS, a computational approach to classifying the lifestyle of phages. Bioinformatics.

[42] Giani T., Arena F., Vaggelli G., Conte V., Chiarelli A., Henrici De Angelis L., Fornaini R., Grazzini M., Niccolini F., Pecile P. (2015). Large Nosocomial Outbreak of Colistin-Resistant, Carbapenemase-Producing *Klebsiella pneumoniae* Traced to Clonal Expansion of an *mgrB* Deletion Mutant. J. Clin. Microbiol..

[43] Chen L., Mathema B., Pitout J.D.D., DeLeo F.R., Kreiswirth B.N. (2014). Epidemic *Klebsiella pneumoniae* ST258 Is a Hybrid Strain. Mbio.

[44] D’Andrea M.M., Amisano F., Giani T., Conte V., Ciacci N., Ambretti S., Santoriello L., Rossolini G.M. (2014). Diversity of capsular polysaccharide gene clusters in KPC-producing *Klebsiella pneumoniae* clinical isolates of Sequence Type 258 involved in the Italian epidemic. PLoS ONE.

[45] Hyman P., Abedon S.T. (2010). Bacteriophage host range and bacterial resistance. Adv. Appl. Microbiol..

[46] Viazis S., Akhtar M., Feirtag J., Brabban A.D., Diez-Gonzalez F. (2011). Isolation and characterization of lytic bacteriophages against enterohaemorrhagic *Escherichia coli*. Appl. Microbiol..

[47] Henry M., Biswas B., Vincent L., Mokashi V., Schuch R., Bishop-Lilly K.A., Sozhamannan S. (2012). Development of a high throughput assay for indirectly measuring phage growth using the OmniLog^™^ system. Bacteriophage.

[48] Luria S.E., Delbrück M. (1943). Mutations of bacteria from virus sensitivity to virus resistance. Genetics.

[49] Zhang C., Liu W., Ren H. (2012). Complete Genome Sequence of Bp7, an *Escherichia coli* Bacteriophage with a Wide Host Range. J. Virol..

[50] Zhang C., Li W., Liu W., Zou L., Yan C., Lu K., Ren H. (2013). T4-Like Phage Bp7, a Potential Antimicrobial Agent for Controlling Drug-Resistant *Escherichia coli* in Chickens. Appl. Environ. Microbiol..

[51] Melegh S., Schneider G., Horváth M., Jakab F., Emődy L., Tigyi Z. (2015). Identification and characterization of CTX-M-15 producing *Klebsiella pneumoniae* clone ST101 in a Hungarian university teaching hospital. Acta Microbiol. Immunol. Hung..

[52] Yao J.D.C., Moellering R.C., Murray P.R., Baron E.J., Pfaller M.A., Tenover F.C., Yolken R.H. (1995). Antimicrobial agents. Manual of Clinical Microbiology.

[53] Pirnay J.P., Blasdel B.G., Bretaudeau L., Buckling A., Chanishvili N., Clark J.R., Corte-Real S., Debarbieux L., Dublanchet A., De Vos D. (2015). Quality and Safety Requirements for Sustainable Phage Therapy Products. Pharm. Res..

[54] Pirnay J.P., De Vos D., Verbeken G., Merabishvili M., Chanishvili N., Vaneechoutte M., Zizi M., Laire G., Lavigne R., Huys I. (2011). The phage therapy paradigm: Prêt-à-porter or sur-mesure?. Pharm. Res..

[55] Montag D., Hashemolhosseini S., Henning U. (1990). Receptor-recognizing proteins of T-even type bacteriophages. The receptor-recognizing area of proteins 37 of phages T4 TuIa and TuIb. J. Mol. Biol..

[56] Chen Z., Schneider T.D. (2005). Information theory based T7-like promoter models: Classification of bacteriophages and differential evolution of promoters and their polymerases. Nucleic Acids Res..

[57] Adriaenssens E.M., Ceyssens P.J., Dunon V., Ackermann H.W., Van Vaerenbergh J., Maes M., de Proft M., Lavigne R. (2011). Bacteriophages LIMElight and LIMEzero of *Pantoea agglomerans*, Belonging to the “phiKMV-Like Viruses”. Appl. Environ. Microbiol..

[58] Ackermann H.W., Krisch H.M., Comeau A.M. (2011). Morphology and genome sequence of phage ϕ1402: A dwarf myovirus of the predatory bacterium *Bdellovibrio bacteriovorus*. Bacteriophage.

[59] Cheepudom J., Lee C.C., Cai B., Meng M. (2015). Isolation, characterization, and complete genome analysis of P1312, a thermostable bacteriophage that infects *Thermobifida fusca*. Front. Microbiol..

[60] Kęsik-Szeloch A., Drulis-Kawa Z., Weber-Dąbrowska B., Kassner J., Majkowska-Skrobek G., Augustyniak D., Lusiak-Szelachowska M., Zaczek M., Górski A., Kropinski A.M. (2013). Characterising the Biology of Novel Lytic Bacteriophages Infecting Multidrug Resistant *Klebsiella pneumoniae*. Virol. J..

[61] Philipson C.W., Voegtly L.J., Lueder M.R., Long K.A., Rice G.K., Frey K.G., Biswas B., Cer R.Z., Hamilton T., Bishop-Lilly K.A. (2018). Characterizing Phage Genomes for Therapeutic Applications. Viruses.

[62] Keen E.C., Bliskovsky V.V., Malagon F., Baker J.D., Prince J.S., Klaus J.S., Adhya S.L. (2017). Novel “superspreader” bacteriophages promote horizontal gene transfer by transformation. MBio.

