# Supplementary material for: Characterization of vB_Kpn_F48, a Newly Discovered Lytic Bacteriophage for Klebsiella pneumoniae of Sequence Type 101
Source: Viruses. 2018 Sep 9;10(9):482. doi: 10.3390/v10090482 (PMC6163469; doi:10.3390/v10090482)
Supplement: Supplementary file 1 [file viruses-10-00482-s001.zip › viruses-353668_Supplementary Figure.pdf]

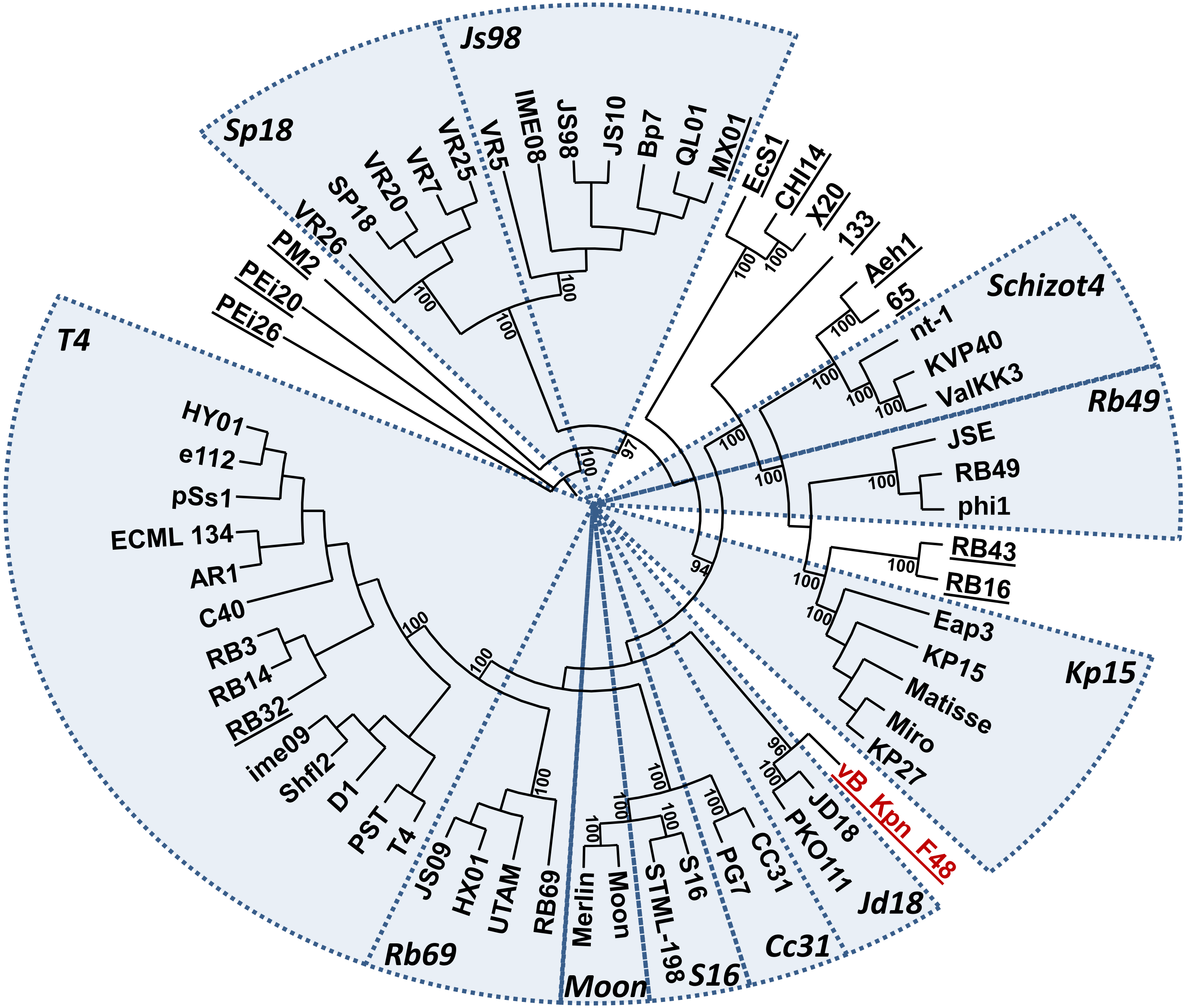



**Figure S1.** Phylogenomic GBDP tree inferred using the formula D4 yielding the maximum average support of 71% among the three different formulas obtained by the VICTOR web server. Distinct genera are highlighted by circular sectors. Phages not assigned to specific genera by the ICTV classification (accessed 19 April 2018) are underlined. GBDP pseudo-bootstrap support values from 100 replicates are shown near branches.
