# Supplementary material for: Characterization of vB_Kpn_F48, a Newly Discovered Lytic Bacteriophage for Klebsiella pneumoniae of Sequence Type 101
Source: Viruses. 2018 Sep 9;10(9):482. doi: 10.3390/v10090482 (PMC6163469; doi:10.3390/v10090482)
Supplement: Supplementary file 1 [file viruses-10-00482-s001.zip › viruses-353668_Supplementary Material.pdf]

Article

Characterization of vB_Kpn_F48, a newly discovered lytic bacteriophage for *Klebsiella pneumoniae* of Sequence Type 101

Nagaia Ciacci[1,2], Marco Maria D'Andrea[1,2], Pasquale Marmo[1], Elisa Demattè[3]•, Francesco Amisano[4]•, Vincenzo Di Pilato[5], Maurizio Fraziano[1], Pietro Lupetti[6], Gian Maria Rossolini[5,7], Maria Cristina Thaller[1]*.

[1] Department of Biology, University of Rome "Tor Vergata", Rome, Italy
[2] Department of Medical Biotechnologies, University of Siena, Siena, Italy
[3] Center for Integrative Biology, CIBIO, University of Trento, Trento, Italy
[4] InBioS - Center for Protein Engineering, Department of Life Sciences, University of Liège, Sart-Tilman, Liège, Belgium
[5] Department of Experimental and Clinical Medicine, University of Florence, Florence, Italy
[6] Department of Life Sciences, University of Siena, Siena, Italy
[7] Clinical Microbiology and Virology Unit, Florence Careggi University Hospital, Florence, Italy
* Correspondence: e-mail: thaller@uniroma2.it, mcthaller@gmail.com; Phone: +39-06-72595984.
•Research was conducted during a training period at the Department of Medical Biotechnologies, University of Siena, Siena, Italy.

Academic Editor: name
Received: date; Accepted: date; Published: date

Supplemental material

Table S1. Primers used for the vB_Kpn_F48 genome characterization. For each primer the nucleotide sequence, the melting temperature (Tm) and the purpose are reported.

Primer Name	Sequence (5' - 3')	Tm (°C)	Purpose
F48_1F	TTAAAGACGGGCAGATGGAC	57.3	PCR/Sequencing
F48_1R	TCCTTCAGGCTGGCTATTTG	57.3	PCR/Sequencing
F48_2F	GACCGTTCAATAAGGCAGAG	57.3	PCR/Sequencing
F48_2R	CATTGTTACTCCTCCTGCTC	57.3	PCR/Sequencing
F48_3F	TCTTCTTCAGAAAGCTCGGC	57.3	PCR/Sequencing
F48_3R	GGGTTGACGGTATCGTTAAC	57.3	PCR/Sequencing
F48_4F	TTTGGAAGCGTGGTTCTAGC	57.3	PCR/Sequencing
F48_4R	AGCTAAGAGTACTGGCGGTA	57.3	PCR/Sequencing
F48_5F	AAACCCCGATTACTTGGGAC	57.3	PCR/Sequencing
F48_5R	GACCAACGTGATGGTGATTG	57.3	PCR/Sequencing
F48_6F	TAGTAGAAGTGTCAACCGCC	57.3	PCR/Sequencing
F48_6R	TTCGCCGAAGATCTGCATAC	57.3	PCR/Sequencing
F48_7F	CATTGAGAGAACCCATTGCG	57.3	PCR/Sequencing
F48_7R	AAGAGGCTCGTTTCCAAGAC	57.3	PCR/Sequencing
F48_8F	TAAGGCTGCTGATACATACCGC	60.2	PCR/Sequencing
F48_8R	ATAAACATCATGGCGTCCGCG	59.8	PCR/Sequencing
F48_9F	TAGGCATCGTTAGCTCCTACG	59.8	PCR/Sequencing
F48_9R	ACGGACAGTAGTTTGGAACGC	59.8	PCR/Sequencing
F48_10F	TAAACCAGGCGGTGACAGAAG	59.8	PCR/Sequencing
F48_10R	ATGGATTAAGATTGGCCCTGTC	58.4	PCR/Sequencing
F48_11F	TAACATTAGCCGTTACACCGC	57.8	PCR/Sequencing
F48_11R	AATGGCGCTGTTGCTTGGAC	59.3	PCR/Sequencing
F48_12F	TCCCTACACCAACAAATGGG	57.3	PCR/Sequencing
F48_12R	AAGTCGTCTTCAAACGCAGGG	59.8	PCR/Sequencing
F48_13F	TTATGCTCAAAGCTGCCGG	56.6	PCR/Sequencing
F48_13R	AGACCCATTTGGCTTAACGC	57.3	PCR/Sequencing
F48_14F	TAAGGACCGACTATGGCTTATAG	58.8	PCR/Sequencing
F48_14R	ATGCTTGCACCTTCTGGAGC	59.3	PCR/Sequencing
F48_TERM_FW	TCAGGTGAAGAGCGTTTGTC	57.3	Direct Sequencing
F48_TERM_REV	TCGGCAGAGCTAATACCAAG	57.3	Direct Sequencing
F48_INT_TERM_FW	TTCATCCGTGCTAAACTGGG	57.3	Direct Sequencing
F48_INT_TERM_REV	CCAGAGCCAAGATACGTTTC	57.3	Direct Sequencing

Table S2. General features of the coding regions of phage vB_Kpn_F48. For each predicted ORF the start and the stop positions, the predicted function of the encoded protein and its closest homolog found in INSDC databases, together with the degree of identity and coverage and the phage from which the ORF derived, are reported. The conserved protein domains detected in some protein, the associated accession numbers and the region in which a particular domain was found, are shown in the last column.

CDS No	Start	Stop	Predicted Function	Closest Protein Homolog	Query cov (%)	ID (%)	Phage	Conserved domains (from-to Accession (short name))
1	69	491	Phage protein	YP_009190702.1	100	74	JD18	
2	540	1109	hypothetical protein	YP_009288809.1	96	24	vB_KpnM_KpV477	
3	1102	1467	hypothetical protein	no significant similarity				
4	1464	1766	Phage protein	YP_009190299.1	96	50	PEi20	
5	1766	2041	Phage protein	YP_009190298.1	98	68	PEi20	
6	2044	2268	Phage protein	YP_009190297.1	100	73	PEi20	1-74 PHA02102 (PHA02102)
7	2278	2514	hypothetical protein	YP_009190296.1	93	68	PEi20	
8	2563	2847	hypothetical protein	YP_009146572.1	51	33	Moon	
9	2837	3034	Phage protein	YP_009190294.1	96	62	PEi20	
10	3165	3383	hypothetical protein	no significant similarity				
11	3380	4342	Phage protein	YP_009190292.1	99	82	PEi20	
12	4733	4864	hypothetical protein	no significant similarity				
13	4923	5144	hypothetical protein	YP_009190290.1	100	57	PEi20	
14	5155	5844	Phage protein	YP_009190289.1	100	85	PEi20	
15	5844	6254	hypothetical protein	no significant similarity				
16	6251	6550	hypothetical protein	no significant similarity				
17	6547	6849	hypothetical protein	YP_009005395.1	100	41	PG7	
18	6846	7292	Nudix hydrolase,	YP_009203850.1	99	67	Merlin	4-86 cd02883 (Nudix_Hydrolase)

			phage-associated					4-62 pfam00293 (NUDIX)<br>8-95 COG0494 (MutT)<br>1-99 PRK10776 (PRK10776)<br>1-79 TIGR00586 (mutt)<br>37-61 PRK11762 (nudE)<br>37-60 TIGR02705 (nudix_YtkD)<br>39-60 COG2816 (NPY1)
19	7324	7818	T4-like phage baseplate hub + tail lysozyme	YP_002854463.1	100	80	RB14	3-161 cd00735 (bacteriophage_T4-like_lysozyme)<br>24-148 pfam00959 (Phage_lysozyme)<br>11-148 COG3772 (RrrD)<br>1-161 PHA02596 (5)
20	7805	8107	Phage protein	YP_009037382.1	100	94	vB_EcoM_JS09	
21	8166	8573	diguanylate cyclase/phosphodiesterase (GGDEF & EAL domains) with PAS/PAC sensor(s)	YP_009203847.1	99	90	Merlin	1-133 pfam03013 (Pyr_excise)
22	8618	9190	Phage protein	YP_009290389.1	100	89	vB_EcoM-UFV13	
23	9328	9681	Phage protein	YP_009324008.1	84	76	MX01	
24	9755	10270	Phage protein	YP_002922458.1	100	51	JS10	45-95 pfam13245 (AAA_19)<br>3-147 PHA02533 (17)
25	10270	10506	hypothetical protein	YP_009203845.1	83	62	Merlin	

26	10508	10738	hypothetical protein	YP_009146562.1	97	56	Moon	
27	10728	10994	Phage protein	YP_009190280.1	100	59	PEi20	
28	11103	11561	Endoribonuclease, RegB protein	YP_004009980.1	97	70	CC31	4-150 pfam10715 (REGB_T4)
29	11571	12110	T4-like phage protein, T4 GC1559	YP_009100652.1	98	73	Shf125875	60-168 cd00254 (LT_GEWL)
30	12460	12924	Phage protein	YP_009146554.1	100	60	Moon	1-154 PHA02595 (tk.4)<br>1-144 cd02901 (Macro_Poa1p_like)<br>4-137 smart00506 (A1pp)<br>21-137 pfam01661 (Macro)<br>20-137 COG2110 (YmdB)
31	12924	13109	hypothetical protein	YP_009190274.1	100	54	PEi20	5-58 pfam04748 (Polysacc_deac_2)
32	13099	13263	hypothetical protein	no significant similarity				
33	13253	13426	hypothetical protein	no significant similarity				
34	13426	14007	Thymidine kinase (EC 2.7.1.21)	YP_009286466.1	98	86	vB_SnwM_CGG4-1	1-188 PRK04296 (PRK04296)<br>1-188 COG1435 (Tdk)<br>2-186 pfam00265 (TK)
35	14175	14387	Phage protein	YP_009190676.1	100	64	JD18	
36	14396	14677	hypothetical protein	YP_009288785.1	100	54	vB_KpnM_KpV477	23-85 PHA02054 (PHA02054)
37	14664	15050	Phage protein	YP_004009967.1	99	69	CC31	1-127 PHA02604 (rl.-1)
38	15141	15407	hypothetical protein	YP_009190268.1	97	52	PEi20	
39	15395	15580	hypothetical protein	YP_009190267.1	93	46	PEi20	
40	15621	15878	Hypothetical	YP_009203821.1	100	82	Merlin	1-70 PRK11019 (PRK11019)

			Zinc-finger containing protein					35-66 TIGR02419 (C4_traR_proteo)<br>40-66 COG1734 (DksA)<br>39-66 pfam01258 (zf-dskA_traR)
41	15875	16156	Phage protein	YP_009146541.1	86	71	Moon	
42	16188	16439	hypothetical protein	YP_009148086.1	85	43	STML-198	
43	16442	16876	hypothetical protein	YP_004009960.1	100	36	CC31	30-102 pfam14126 (DUF42939)<br>33-105 TIGR04286 (MSEP-CTERM)
44	16873	17106	hypothetical protein	YP_009190666.1	89	42	JD18	
45	17108	17629	hypothetical protein	YP_009190260.1	99	35	PEi20	
46	17626	18636	Thioredoxin, phage-associated	YP_009190259.1	100	85	PEi20	1-336 PHA02603 (nrdC.11)<br>10-149 pfam10127 (Nuc-transf)
47	18694	18954	hypothetical protein	YP_009286454.1	89	36	vB_SnwM_CGG4-1	
48	19047	19199	hypothetical protein	no significant similarity				
49	19207	19665	hypothetical protein	YP_009190253.1	100	67	PEi20	
50	19665	19841	hypothetical protein	no significant similarity				
51	19841	20158	hypothetical protein	YP_009323290.1	63	32	WG01	
52	20291	20142	hypothetical protein	no significant similarity				
53	20593	21366	hypothetical protein	YP_009203806.1	100	69	Merlin	
54	21366	21599	hypothetical protein	no significant similarity				
55	21596	22030	hypothetical protein	YP_004009942.1	95	79	CC31	
56	22147	22314	hypothetical protein	no significant similarity				
57	22325	23272	hypothetical protein	YP_009190247.1	80	23	PEi20	

58	23269	23493	Thioredoxin, phage-associated	YP_009190650.1	100	71	JD18	
59	23490	23804	hypothetical protein	YP_009190649.1	100	82	JD18	
60	23801	24307	Phage protein	YP_009289473.1	98	45	PKO111	
61	24307	24606	hypothetical protein	YP_009205777.1	52	58	vB_EcoM_VR5	
62	24584	24847	Thioredoxin, phage-associated	YP_009289200.1	100	74	SHSML-52-1	14-84 cd03418 (GRX_GRXb_1_3_like)<br>14-84 TIGR02181 (GRX_bact)<br>14-84 PRK10638 (PRK10638)<br>14-76 pfam00462 (Glutaredoxin)<br>14-84 COG0695 (GrxC)<br>14-84 PRK12759 (PRK12759)
63	24844	25011	hypothetical protein	YP_009202812.1	96	45	QL01	
64	25008	25196	hypothetical protein	YP_008770398.1	58	44	Spock	
65	25193	25510	Phage protein	YP_009190240.1	100	61	PEi20	
66	25627	26067	Pin protease inhibitor	YP_009190238.1	100	34	PEi20	2-139 pfam10465 (Inhibitor_I24)
67	26103	26576	Phage endonuclease #T4-like phage gp49, endonuclease VII #T4 GC1525	YP_009213913.1	100	75	vB_EcoM_VR26	1-97 pfam02945 (Endonuclease_7)<br>105-157 pfam09124 (Endonuc-dimeris)<br>1-157 PHA02565 (49)
68	26692	26892	hypothetical protein	no significant similarity				
69	26889	27194	Glutaredoxin	YP_009190630.1	98	61	JD18	3-79 pfam00462 (Glutaredoxin)<br>15-79 cd02066 (GRX_family)<br>1-79 COG0695 (GrxC)

								1-88 PRK11200 (grxA)<br>3-92 TIGR02183 (GRXA)
70	27334	27513	hypothetical protein	YP_009190232.1	98	52	PEi20	
71	27513	27770	hypothetical protein	YP_009190231.1	97	30	PEi20	
72	27770	28054	Phage protein	YP_009195480.1	100	50	vB_PmiM_Pm5461	
73	28128	28460	Phage protein	YP_009146502.1	100	76	Moon	
74	28457	28738	hypothetical protein	YP_009148051.1	74	36	STML-198	
75	28731	28958	hypothetical protein	YP_004009918.1	90	54	CC31	
76	28955	29221	hypothetical protein	YP_009190226.1	98	60	PEi20	
77	29288	29827	T4-like phage RNA polymerase sigma factor for late transcription	YP_009190621.1	100	86	JD18	1-179 PHA02547 (55)
78	29850	30041	Phage protein	YP_009190620.1	98	68	JD18	
79	30043	30387	Phage protein	YP_009288733.1	84	61	vB_KpnM_KpV477	18-114 PHA02571 (a-gt.4)<br>38-107 pfam10849 (DUF2654)
80	30365	30562	Phage protein	YP_009190221.1	100	71	PEi20	
81	30648	30833	hypothetical protein	YP_009146494.1	100	69	Moon	2-44 PHA00911 (21)
82	30898	31920	Phage recombination-related endonuclease Gp47	YP_009289495.1	99	81	PKO111	2-87 cd00840 (MPP_Mre11_N)<br>1-116 TIGR00619 (sbcd)<br>1-340 PHA02546 (47)<br>1-340 COG0420 (SbcD)<br>1-104 pfam00149 (Metallophos)

83	31917	33599	Phage recombination-relate d endonuclease Gp46	YP_009286413.1	100	77	vB_SnwM_CGG4-1	1-138 cd03279 (ABC_sbcCD)<br>9-199 pfam13476 (AAA_23)<br>433-552 cd03279 (ABC_sbcCD)<br>4-560 PHA02562 (46)<br>5-391 COG0419 (SbcC)<br>1-409 TIGR00618 (sbcc)<br>21-403 pfam02463 (SMC_N)
84	33596	33793	Phage protein	YP_009202786.1	95	63	QL01	
85	33815	34189	RNA polymerase, phage-associated	YP_009190611.1	99	71	JD18	7-117 pfam10789 (Phage_RpbA)
86	34208	34912	Sliding clamp DNA polymerase accessory protein, phage associated # T4-like phage gp45 #T4 GC0153	YP_009190610.1	100	62	JD18	115-226 pfam09116 (gp45-slide_C)<br>2-103 pfam02916 (DNA_PPF)<br>1-232 PHA02545 (45)
87	34958	35917	Replication factor C small subunit / Phage DNA polymerase clamp loader subunit	YP_009289500.1	99	80	PKO111	52-158 pfam00004 (AAA)<br>27-155 cd00009 (AAA)<br>1-319 PHA02544 (44)<br>16-261 COG2256 (RarA)<br>30-196 TIGR00678 (holB)<br>30-157 pfam13177 (DNA_pol3_delta2)<br>52-148 smart00382 (AAA)

88	35908	36486	Phage DNA polymerase clamp loader subunit Gp62	YP_009289501.1	96	66	PKO111	3-192 PHA02593 (62)<br>41-178 pfam16790 (Phage_clamp_A)
89	36480	36857	Phage endoribonulcease translational repressor of early genes, regA	YP_009146484.1	100	88	Moon	1-125 PHA02543 (regA)<br>4-124 pfam01818 (Translat_reg)
90	36942	39647	DNA polymerase (EC 2.7.7.7), phage-associated	YP_007235888.1	100	74	phiR1-RT	5-501 PHA02524 (43A)<br>519-899 PHA02523 (43B)<br>387-779 cd00145 (POLBc)<br>109-333 cd05160 (DEDDy_DNA_polB_exo)<br>1-900 PHA02528 (43)<br>111-624 smart00486 (POLBc)<br>388-857 pfam00136 (DNA_polB)<br>145-772 COG0417 (PolB)<br>389-650 TIGR00592 (pol2)
91	39741	41405	Phage protein	YP_002922391.1	99	81	JS10	4-101 cd04181 (NTP_transferase)<br>351-412 cd05153 (HomoserineK_II)<br>3-77 COG1213 (COG1213)<br>3-94 pfam00483 (NTP_transferase)<br>369-419 COG4857 (COG4857)<br>4-70 TIGR03310 (matur_MocA_YgfJ)<br>364-414 smart00587 (CHK)

								369-407 PRK09550 (mtnK)<br>1-108 COG1208 (GCD1)<br>3-115 TIGR03992 (Arch_glmU)<br>350-416 pfam01636 (APH)<br>3-95 PRK14357 (glmU)
92	41441	41569	hypothetical protein	no significant similarity				
93	41523	41882	Phage protein	YP_009323940.1	100	87	MX01	3-108 TIGR01689 (EcbF-BcbF)
94	41879	42418	Phage protein	YP_001595169.1	98	78	JS98	4-98 cd01672 (TMPK)<br>1-140 COG0125 (Tmk)<br>1-33 PRK00698 (tmk)
95	42415	43575	Phage protein	YP_009190202.1	99	90	PEi20	55-360 COG0826 (PrtC)<br>95-308 pfam01136 (Peptidase_U32)
96	43572	43817	hypothetical protein	YP_009190201.1	96	73	PEi20	10-78 pfam01817 (CM_2)<br>10-78 smart00830 (CM_2)<br>5-78 COG1605 (PheA)<br>6-78 PRK07248 (PRK07248)<br>6-78 TIGR01791 (CM_archaeal)<br>1-77 PRK12595 (PRK12595)
97	43796	44509	Thymidylate synthase (EC 2.1.1.45)	YP_001595168.1	97	87	JS98	13-237 PRK00956 (thyA)<br>49-234 cd00351 (TS_Pyrimidine_Hmase)<br>10-233 TIGR03283 (thy_syn_methano)<br>121-234 COG0207 (ThyA)<br>123-234 pfam00303 (Thymidylat_synt)

98	44509	45408	Phage protein	YP_009190199.1	100	70	PEi20	
99	45483	45752	hypothetical protein	no significant similarity				
100	45836	47005	Phage recombination protein	YP_009211461.1	98	93	PM2	39-292 cd00983 (recA)<br>39-250 pfam00154 (RecA)<br>39-302 TIGR02012 (tigrfam_recA)<br>39-260 PRK09519 (recA)<br>9-279 COG0468 (RecA)
101	46995	47336	Phage capsid vertex assembly chaperone	YP_009190599.1	99	54	JD18	49-101 pfam11113 (Phage_head_chap)
102	47346	48779	DNA primase/helicase, phage-associated	YP_009288713.1	100	80	vB_KpnM_KpV477	179-382 cd00984 (DnaB_C)<br>179-360 pfam13481 (AAA_25)<br>1-477 PHA02542 (41)<br>36-382 COG0305 (DnaB)<br>5-385 TIGR03600 (phage_DnaB)<br>179-339 pfam03796 (DnaB_C)
103	48840	49121	hypothetical protein	YP_004009892.1	100	51	CC31	
104	49118	49396	hypothetical protein	YP_009190191.1	100	43	PEi20	
105	49421	49588	Phage protein	YP_009190593.1	72	73	JD18	3-48 PHA02541 (23)<br>3-48 pfam07068 (Gp23)
106	49612	50631	DNA primase (EC 2.7.7.-) / DNA helicase (EC 3.6.1.-), phage-associated	YP_009288708.1	100	71	vB_KpnM_KpV477	175-204 pfam08275 (Toprim_N)<br>3-339 PHA02540 (61)<br>175-247 TIGR01391 (dnaG)<br>175-311 COG0358 (DnaG)

107	50857	51138	hypothetical protein	YP_009190186.1	98	68	PEi20	2-51 cd11536 (NTP-PPase_iMazG)
108	51201	51953	Phage protein	YP_007010327.1	49	39	CC2	1-82 COG3755 (YecT)<br>26-95 pfam07007 (DUF1311)<br>1-104 COG4461 (Lprl)<br>5-127 PHA02067 (PHA02067)
109	51997	52515	dCTP pyrophosphatase (EC 3.6.1.12), phage-associated	YP_009202758.1	100	87	QL01	1-172 PHA02602 (56)<br>69-172 cd11527 (NTP-PPase_dUTPase)<br>129-172 pfam08761 (dUTPase_2)
110	52515	52730	hypothetical protein	YP_009288700.1	57	85	vB_KpnM_KpV477	
111	52754	52990	Phage capsid and scaffold	YP_009288699.1	100	83	vB_KpnM_KpV477	2-57 pfam16855 (Soc)
112	53070	53309	hypothetical protein	YP_009190182.1	89	54	PEi20	
113	53306	53479	hypothetical protein	no significant similarity				
114	53476	53871	Phage protein	YP_009190181.1	82	38	PEi20	
115	54039	54542	hypothetical protein	YP_009288697.1	100	35	vB_KpnM_KpV477	
116	54542	54718	Molybdenum ABC transporter, periplasmic molybdenum-binding protein ModA (TC 3.A.1.8.1)	YP_001595147.1	98	67	JS98	
117	54786	55388	NAD--protein	YP_009190178.1	98	72	PEi20	

			ADP-ribosyltransferase modA (EC 2.4.2.-)					
118	55457	55648	hypothetical protein	YP_009210230.1	57	50	slur02	5-48 pfam10493 (Rod_C)
119	55707	56450	Phage anti-termination	YP_004009877.1	97	44	CC31	
120	56450	56755	hypothetical protein	YP_007501056.1	99	42	vB_SenMS16	
121	56752	58080	DNA helicase (EC 3.6.1.-), phage-associated	YP_009005281.1	99	75	PG7	6-196 pfam13604 (AAA_30)<br>9-143 cd00009 (AAA)<br>395-442 pfam13538 (UvrD_C_2)<br>34-57 PRK13768 (PRK13768)<br>11-195 TIGR01448 (recD_rel)<br>32-263 COG0507 (RecD)<br>34-156 PRK10875 (recD)<br>7-119 smart00487 (DEXDc)<br>7-68 pfam13086 (AAA_11)
122	58088	58309	hypothetical protein	YP_009190174.1	100	45	PEi20	
123	58306	58989	T4-like phage DexA exonuclease A	YP_009211433.1	100	68	PM2	1-227 PHA02570 (dexA)
124	58986	59159	hypothetical protein	YP_009190856.1	94	74	JD18	
125	59169	59549	Transcriptional regulator	YP_009190171.1	97	41	PEi20	
126	59610	59831	cef modifier of suppressor tRNAs,	NP_861698.1	91	78	RB69	

			phage-associated					
127	59833	60546	hypothetical protein	YP_004009870.1	33	35	CC31	147-183 pfam04108 (APG17)
128	60533	60709	hypothetical protein	YP_004009868.1	100	72	CC31	1-58 COG2331 (COG23319)<br>1-52 TIGR02605 (CxxC_CxxC_SSSS)<br>1-44 smart00834 (CxxC_CxxC_SSSS)<br>1-31 pfam09723 (Zn-ribbon_8)<br>3-27 TIGR03680 (eif2g_arch)
129	60867	62708	Phage DNA topoisomerase large subunit (EC 5.99.1.3)	YP_009190163.1	99	85	PEi20	417-523 cd01030 (TOPRIM_TopoIIA_like)<br>261-347 cd03481 (TopoIIA_Trans_ScTopoIIA)<br>193-368 pfam00204 (DNA_gyraseB)<br>51-165 cd00075 (HATPase_c)<br>47-149 pfam02518 (HATPase_c)<br>47-165 smart00387 (HATPase_c)<br>525-612 pfam16898 (TOPRIM_C)<br>3-611 PHA02569 (39)<br>47-611 smart00433 (TOP2c)<br>6-611 COG0187 (GyrB)<br>4-613 TIGR01058 (parE_Gpos)
130	62738	63061	hypothetical protein	YP_009202734.1	97	56	QL01	
131	63048	63251	Phage rIIA lysis inhibitor	YP_009146435.1	98	67	Moon	
132	63260	65407	Phage rIIA lysis inhibitor	YP_009289531.1	99	46	PKO111	39-136 smart00387 (HATPase_c)<br>38-136 pfam02518 (HATPase_c)

								41-136 cd00075 (HATPase_c)<br>37-145 TIGR01052 (top6b)
133	65420	66325	Phage rIIB lysis inhibitor	YP_009289532.1	99	82	PKO111	
134	66366	66557	Phage protein	YP_009190458.1	100	78	PEi20	
135	66557	66841	Phage protein	YP_007004180.1	87	78	Bp7	
136	66906	67391	Phage endonuclease	YP_003934906.1	97	51	SP18	48-114 PHA02151 (PHA02151)
137	67439	67678	hypothetical protein	no significant similarity				
138	67726	67845	hypothetical protein	YP_009190454.1	89	54	PEi20	
139	68021	68479	Phage protein	YP_009005552.1	96	67	PG7	8-152 pfam06591 (Phage_T4_Ndd)
140	68715	68828	hypothetical protein	YP_009205956.1	97	44	vB_EcoM_VR5	
141	68821	68961	hypothetical protein	YP_009288944.1	100	63	vB_KpnM_KpV477	1-46 TIGR01597 (PYST-B)
142	68967	70331	DNA topoisomerase, phage-associated	YP_009190447.1	96	75	PEi20	40-450 cd00187 (TOP4c)<br>15-454 PHA02592 (52)<br>19-454 smart00434 (TOP4c)<br>40-454 pfam00521 (DNA_topoisoIV)<br>8-454 COG0188 (GyrA)<br>20-426 TIGR01063 (gyrA)
143	70328	70507	hypothetical protein	no significant similarity				
144	70757	71404	hypothetical protein	YP_009190445.1	100	64	PEi20	70-117 cd04268 (ZnMc_MMP_like)
145	71479	72111	Phage transcriptional regulator of middle promoters	YP_009190443.1	100	73	PEi20	2-97 pfam09114 (MotA_activ)<br>111-206 pfam09158 (MotCF)

146	72119	72304	hypothetical protein	YP_009190442.1	98	50	PEi20	
147	72306	72629	Phage anti-restriction nuclease	YP_002854592.1	100	66	RB14	
148	72626	72805	hypothetical protein	YP_009056803.1	84	60	vB_EcoM_PhAPE C2	
149	72996	73457	Phage anti-restriction nuclease	YP_009207447.1	99	86	vB_EcoM_VR20	
150	73457	73573	hypothetical protein	YP_009288935.1	97	89	vB_KpnM_KpV477	
151	73566	73826	hypothetical protein	YP_009146715.1	100	47	Moon	30-74 cd03015 (PRX_Typ2cys)<br>44-85 TIGR04141 (TIGR04141)
152	73826	74131	hypothetical protein	no significant similarity				
153	74185	74457	Phage anti-sigma factor	YP_004010118.1	97	65	CC31	2-81 pfam09010 (AsiA)
154	75114	74458	Phage holin	YP_009190823.1	100	69	JD18	4-217 pfam11031 (Phage_holin_T)
155	75535	75134	hypothetical protein	YP_009190822.1	100	51	JD18	
156	79780	75569	Phage tail fiber protein	YP_003580110.1	88	63	KP15	1095-1286 PHA02584 (34)
157	80467	79790	Phage tail connector protein	YP_009289546.1	100	86	PKO111	1-225 pfam03903 (Phage_T4_gp36)
158	81645	80524	Phage tail connector protein	YP_009288926.1	99	51	vB_KpnM_KpV477	
159	85490	81657	Phage long tail fiber proximal subunit	YP_009289548.1	100	50	PKO111	1-1275 PHA02584 (34)

160	85576	86517	Phage ribonuclease H (EC 3.1.26.4)	YP_009205940.1	99	76	vB_EcoM_VR5	15-188 cd09860 (PIN_T4-like)<br>187-312 pfam09293 (RNaseH_C)<br>188-266 cd09899 (H3TH_T4-like)<br>1-312 PHA02567 (rnh)<br>51-210 smart00475 (53EXOc)<br>51-181 pfam02739 (5_3_exonuc_N)<br>57-210 TIGR00593 (pola)<br>6-210 COG0258 (Exo)
161	86528	86797	Phage double-stranded DNA binding protein #T4-like dsbA, late transcriptional regulation #T4 GC1668	YP_009190426.1	100	64	PEi20	1-88 PHA02599 (dsbA)<br>24-89 pfam11126 (Phage_DsbA)<br>17-85 cd08645 (FMT_core_GART)
162	86778	87071	Transcriptional regulator	YP_009288922.1	91	66	vB_KpnM_KpV477	22-93 pfam16805 (Trans_coact)
163	87068	87724	Phage DNA helicase loader	YP_009190814.1	99	87	JD18	14-106 pfam08993 (T4_Gp59_N)<br>113-216 pfam08994 (T4_Gp59_C)<br>1-217 PHA02559 (59)
164	87801	88697	Single stranded DNA-binding protein,	YP_004010107.1	100	67	CC31	1-279 PHA02550 (32)<br>28-116 pfam08804 (gp32)<br>149-208 PRK06599 (PRK06599)

			phage-associated					
165	88800	89021	hypothetical protein	YP_009146696.1	94	41	Moon	
166	89022	89402	Phage protein	YP_009190420.1	95	46	PEi20	30-126 pfam03197 (FRD2)
167	89458	89736	Phage protein	YP_009146693.1	85	81	Moon	
168	89815	90135	hypothetical protein	YP_009289557.1	100	96	PKO111	
169	90218	90823	Dihydrofolate reductase, phage-associated	YP_009190412.1	97	62	PEi20	22-151 cd00209 (DHFR)<br>23-151 pfam00186 (DHFR_1)<br>1-142 COG0262 (FolA)<br>23-151 PRK10769 (folA)<br>23-186 PTZ00164 (PTZ00164)
170	90816	91139	hypothetical protein	YP_009146687.1	99	41	Moon	
171	91120	91473	hypothetical protein	YP_009005517.1	95	54	PG7	
172	91556	91684	hypothetical protein	YP_009111049.1	100	71	pSs-1	
173	91684	92544	Thymidylate synthase (EC 2.1.1.45)	YP_009324126.1	100	83	MX01	2-286 pfam00303 (Thymidylat_synt)<br>1-286 PRK01827 (thyA)<br>3-286 TIGR03284 (thym_sym)<br>1-286 COG0207 (ThyA)<br>3-227 cd00351 (TS_Pyrimidine_HMase)<br>3-286 PTZ00164 (PTZ00164)
174	92541	92765	hypothetical protein	YP_009190408.1	100	77	PEi20	
175	92816	92968	hypothetical protein	YP_009190803.1	100	74	JD18	
176	93031	93264	Phage protein	YP_004010093.1	100	79	CC31	2-62 pfam14216 (DUF4326)
177	93261	95516	Ribonucleotide	YP_009190406.1	100	82	PEi20	164-727 cd01679 (RNR_1)

			reductase of class Ia (aerobic), alpha subunit (EC 1.17.4.1)					136-214 pfam00317 (Ribonuc_red_lgN)<br>1-87 pfam03477 (ATP-cone)<br>1-751 PHA02572 (ntdA)<br>140-728 TIGR02506 (NrdA_NrdA)<br>57-750 COG0209 (NrdA)<br>217-724 pfam02867 (Ribonuc_red_lgC)
178	95555	95938	hypothetical protein	no significant similarity				
179	95989	97131	Ribonucleotide reductase of class Ia (aerobic), beta subunit (EC 1.17.4.1)	YP_009190800.1	100	84	JD18	3-371 PRK09101 (nrdB)<br>1-371 COG0208 (NrdF)<br>28-343 cd01049 (RNRR2)<br>32-323 pfam00268 (Ribonuc_red_sm)<br>41-157 TIGR04171 (RNR_1b_NrdF)<br>38-343 PRK12759 (PRK12759)
180	97159	97575	Phage endonuclease	YP_009289564.1	100	83	PKO111	4-137 PHA02598 (denA)<br>35-133 cd10436 (GIY-YIG_EndoII_Hpy188I_like)<br>33-134 smart00465 (GIYc)<br>34-62 pfam01541 (GIY-YIG)<br>42-131 PRK12306 (uvrC)
181	97572	98729	RNA ligase, phage-associated	YP_009288910.1	100	56	vB_KpnM_KpV477	1-376 PHA02589 (rnlA)<br>3-376 TIGR02308 (RNA_lig_T4_1)<br>53-248 pfam09511 (RNA_lig_T4_1)<br>54-123 COG5324 (Trl1)

182	98788	99297	Phage alc transcription terminator	YP_009190400.1	98	63	PEi20	
183	99387	99635	hypothetical protein	YP_009146676.1	93	32	Moon	
184	99701	99925	Phage outer membrane lipoprotein Rz1	YP_007236059.1	94	60	phiR1-RT	
185	99970	100455	hypothetical protein	YP_009190397.1	99	53	PEi20	
186	100455	100664	hypothetical protein	YP_006906137.1	100	48	NJ01	
187	100661	100837	hypothetical protein	no significant similarity				
188	100834	101736	3'-phosphatase, 5'-polynucleotide kinase, phage-associated	YP_009148660.1	100	76	HY01	1-139 COG4639 (COG4639)<br>4-149 TIGR03574 (selen_PSTK)<br>161-213 cd01427 (HAD_like)<br>1-25 PRK08118 (PRK08118)<br>4-59 pfam13207 (AAA_17)<br>156-234 pfam03767 (Acid_phosphat_B)<br>162-231 smart00775 (LNS2)<br>1-300 PHA02530 (pseT)<br>4-139 pfam13671 (AAA_33)<br>4-139 TIGR04075 (bacter_Pnkp)<br>1-149 COG4088 (Kti12)
189	101766	102029	hypothetical protein	no significant similarity				
190	102088	102303	Phage protein	YP_009286570.1	100	76	vB_SnwM_CGG4-	

							1	
191	102313	102591	hypothetical protein	YP_009190791.1	98	44	JD18	
192	102581	102811	hypothetical protein	YP_009190392.1	97	54	PEi20	
193	102855	103847	2-keto-3-deoxy-D-arabino-heptulosonate-7-phosphate synthase I alpha (EC 2.5.1.54)	YP_009056797.1	99	68	vB_EcoM_PhAPEC2	5-329 PRK09261 (PRK09261)<br>5-326 COG0722 (AroG1)<br>5-328 TIGR00034 (aroFGH)<br>37-324 pfam00793 (DAHP_synth_1)
194	103850	104410	dCMP deaminase (EC 3.5.4.12); Late competence protein ComEB	YP_009190390.1	100	87	PEi20	1-182 PHA02588 (cd)<br>3-157 cd01286 (deoxycytidylate_deaminase)<br>1-164 COG2131 (ComEB)<br>10-178 TIGR02571 (ComEB)<br>2-143 pfam00383 (dCMP_cyt_deam_1)<br>54-164 TIGR00326 (eubact_ribD)<br>54-143 PRK10786 (ribD)
195	104410	104745	hypothetical protein	no significant similarity				
196	104742	105065	Phage tail fibers	YP_009190388.1	99	82	PEi20	22-105 pfam10902 (DUF2693)
197	105130	105459	Phage head assembly chaperone protein	YP_009146662.1	100	69	Moon	5-108 pfam00166 (Cpn10)<br>5-65 cd00320 (cpn10)
198	105565	105813	Phage rIII lysis inhibitor accessory	YP_004009824.1	100	74	Acj61	
199	106182	106358	Phage protein	YP_009288895.1	100	98	vB_KpnM_KpV477	
200	106465	106833	Phage protein	YP_009146659.1	97	78	Moon	3-121 pfam06019 (Phage_30_8)
201	106873	107403	hypothetical protein	YP_004010067.1	56	32	CC31	

202	107413	107889	hypothetical protein	no significant similarity				
203	107952	108308	Phage protein	YP_004010066.1	100	77	CC31	1-118 pfam06919 (Phage_T4_Gp30_7)
204	108347	108826	hypothetical protein	no significant similarity				
205	108823	109104	hypothetical protein	YP_009288892.1	100	57	vB_KpnM_KpV477	
206	109115	109279	hypothetical protein	YP_009190379.1	100	59	PEi20	6-42 PRK15101 (PRK15101)
207	109276	109458	hypothetical protein	no significant similarity				
208	109448	109660	hypothetical protein	YP_009190776.1	92	52	JD18	
209	109638	110480	Phage protein	YP_009288888.1	96	48	vB_KpnM_KpV477	1-196 PHA02597 (30,2)<br>1-116 COG1011 (YigB)
210	110488	110763	Phage protein	NP_861903.1	100	64	RB69	1-82 pfam11243 (DUF3045)
211	110760	112238	DNA ligase, phage-associated	YP_007004245.1	99	67	Bp7	135-361 pfam01068 (DNA_ligase_A_M)<br>134-361 cd06846 (Adenylation_DNA_ligase_like)<br>1-490 PHA02587 (39)<br>159-489 COG1793 (CDC9)<br>136-486 TIGR00574 (dnl1)
212	112301	114394	RNA polymerase-ADP-ribosyltransferase Alt	YP_009190371.1	100	60	PEi20	387-624 cd00233 (VIP2)<br>416-626 pfam03496 (ADPrib_exo_Tox)<br>458-635 PHA02057 (PHA02057)<br>1-696 PHA02566 (alt)
213	114455	114742	Phage protein	YP_009180694.1	100	49	slur14	
214	115690	114773	Phage baseplate tail tube initiator	YP_009289587.1	100	72	PKO111	1-305 PHA02605 (54)<br>144-246 pfam06841 (Phage_T4_gp19)

215	116745	115690	Phage baseplate tail tube cap (T4-like gp48)	YP_009190767.1	99	77	JD18	1-349 PHA02613 (48)<br>13-343 pfam11091 (T4_tail_cap)
216	118455	116755	Phage baseplate hub	YP_009289589.1	97	43	PKO111	357-516 PTZ00121 (PTZ00121)
217	118982	118455	Phage baseplate hub	YP_009190765.1	100	65	JD18	21-174 pfam11110 (Phage_hub_GP28)
218	120096	118954	Phage baseplate hub subunit	YP_009289591.1	100	72	PKO111	4-198 pfam09097 (Phage-tail_1)<br>201-373 pfam09096 (Phage-tail_2)<br>4-371 PHA02612 (27)
219	120851	120093	Phage baseplate	YP_009190763.1	98	63	JD18	78-202 pfam12322 (T4_baseplate)<br>1-251 PHA02611 (51)
220	120902	121528	Phage baseplate hub assembly chaperone (T4-like gp26)	YP_009288876.1	99	64	vB_KpnM_KpV477	84-205 pfam12322 (T4_baseplate)<br>20-207 PHA02611 (51)
221	121525	121914	Phage baseplate wedge subunit (T4-like gp25)	YP_009190761.1	98	72	JD18	1-129 PHA00415 (25)<br>27-116 pfam04965 (GPW_gp25)<br>31-99 COG3628 (COG3628)<br>29-96 TIGR03357 (VI_zyme)
222	121962	122381	Single stranded DNA-binding protein, phage-associated	YP_009190760.1	98	79	JD18	9-138 pfam11056 (UvsY)
223	122412	122576	Phage protein	YP_009288873.1	94	75	vB_KpnM_KpV477	4-54 PHA02610 (uvsY.-2)<br>4-53 pfam10886 (DUF2685)

224	122865	122638	DNA helicase, phage-associated	YP_009005466.1	97	70	PG7	3-75 PHA02609 (uvsW.1)<br>19-63 pfam11637 (UvsW)
225	124374	122875	DNA helicase, phage-associated	YP_009289598.1	99	79	PKO111	111-256 pfam04851 (ResIII)<br>318-448 cd00079 (HELICc)<br>357-440 smart00490 (HELICc)<br>129-258 cd00046 (DEXDc)<br>341-438 pfam00271 (Helicase_C)<br>1-499 PHA02558 (uvsW)<br>117-453 COG1061 (SSL2)<br>112-284 smart00487 (DEXDc)
226	124432	125151	Inh inhibitor of gp21 prohead protease	YP_009324076.1	100	59	MX01	
227	125161	125892	Phage capsid and scaffold	YP_009288869.1	95	91	vB_KpnM_KpV477	
228	126096	126296	Phage capsid and scaffold	YP_003734323.1	100	82	IME08	
229	126397	126615	Phage protein	YP_009190355.1	100	88	PEi20	1-57 pfam11242 (DUF2774)
230	126608	126970	hypothetical protein	YP_009190354.1	100	74	PEi20	
231	127118	126960	hypothetical protein	no significant similarity				
232	127086	128084	RNA ligase, phage-associated	YP_009190353.1	100	77	PEi20	2-331 TIGR02307 (RNA_lig_RNL2)<br>31-224 pfam09414 (RNA_ligase)<br>35-163 cd07894 (Adenylation_RNA_ligase)<br>90-231 TIGR02306 (RNA_lig_DRB0094)

233	128453	128629	hypothetical protein	YP_009289603.1	100	63	PKO111	
234	129919	128651	Phage capsid vertex	YP_009207368.1	99	80	vB_EcoM_VR20	2-422 PHA02548 (24)<br>114-383 pfam07068 (Gp23)
235	131190	129916	Phage capsid vertex	YP_009202910.1	99	74	QL01	1-422 PHA02548 (24)<br>29-382 pfam07068 (Gp23)
236	132848	131283	Phage major capsid protein	YP_009005455.1	99	86	PG7	1-521 PHA02541 (23)<br>6-501 pfam07068 (Gp23)
237	133687	132869	Phage prohead assembly (scaffolding) protein	YP_009288861.1	100	72	vB_KpnM_KpV477	1-272 PHA02557 (22)
238	134381	133716	Phage prohead assembly (scaffolding) protein	YP_009190746.1	98	79	JD18	7-220 PHA00911 (21)<br>8-195 pfam03420 (Peptidase_S77)
239	134806	134381	Phage capsid and scaffold	YP_009190745.1	100	77	JD18	1-140 PHA02586 (68)
240	135048	134806	Phage prohead core protein	YP_009209927.1	70	63	vB_EcoM_VR25	1-45 PHA02608 (65)
241	136617	135049	Phage portal vertex of the head	YP_009190743.1	100	81	JD18	2-517 PHA02531 (20)<br>6-507 pfam07230 (Peptidase_S80)
242	137208	136717	Phage tail fibers	YP_009288856.1	99	87	vB_KpnM_KpV477	1-162 PHA02551 (19)<br>17-162 pfam06841 (Phage_T4_gp19)
243	139371	137389	Phage tail sheath monomer	YP_009289428.1	99	81	PKO111	2-646 pfam04984 (Phage_sheath_1)<br>405-646 COG3497 (COG3497)

								1-657 PHA02539 (18)
244	141240	139405	Phage terminase, large subunit	YP_009190343.1	100	92	PEi20	142-288 pfam05876 (Terminase_GpA)<br>80-609 PHA02533 (17)<br>158-573 pfam03237 (Terminase_6)
245	141707	141240	Phage terminase, small subunit	YP_009203902.1	100	86	Merlin	1-151 PHA02585 (16)<br>3-144 pfam11053 (DNA_Packaging)
246	142497	141718	Proximal tail sheath stabilization protein	YP_009190738.1	98	73	JD18	1-259 PHA02556 (15)<br>1-233 pfam16724 (T4-gp15_tss)
247	143151	142558	Phage-associated homing endonuclease	YP_009288851.1	97	84	vB_KpnM_KpV477	111-193 cd00283 (GIY-YIG_Cterm)
248	143940	143161	T4-like phage head completion, neck hetero-dimeric protein (T4-like gp14)	YP_009190340.1	99	77	PEi20	25-235 PHA02555 (14)<br>21-254 pfam11649 (T4_neck-protein)
249	144888	143944	T4-like phage head completion, neck hetero-dimeric protein (T4-like gp13)	YP_009190736.1	97	79	JD18	6-314 PHA02554 (13)
250	146716	144950	Phage neck whiskers	YP_009289422.1	99	40	PKO111	383-448 pfam07921 (Fibritin_C)<br>60-145 smart00150 (SPEC)<br>3-451 PHA02607 (wac)<br>43-285 COG1196 (Smc)

251	148081	146726	Phage straight tail fiber (short tail fiber)	YP_009190734.1	100	56	JD18	52-264 PHA02584 (34)
252	148734	148081	Phage baseplate wedge subunit and tail pin (T4-like gp11) # T4 GC 1597	YP_009289420.1	99	47	PKO111	4-217 PHA02583 (11)<br>4-217 pfam08677 (GP11)
253	150545	148734	Phage baseplate wedge subunit and tail pin (T4-like gp10)	YP_009288845.1	99	64	vB_KpnM_KpV477	1-603 PHA02582 (10)<br>2-263 pfam07880 (T4_gp9_10)
254	151432	150548	Phage baseplate wedge tail fiber connector (T4-like gp9)	YP_009190731.1	98	44	JD18	5-291 PHA02581 (9)<br>8-280 pfam07880 (T4_gp9_10)
255	152528	151497	Phage baseplate wedge subunit (T4-like gp8)	YP_009289417.1	100	72	PKO111	10-343 PHA02580 (8)<br>16-343 pfam09215 (Phage-Gp8)
256	155636	152529	Phage baseplate wedge initiator (T4-like gp7)	YP_009289416.1	100	69	PKO111	1-1035 PHA02579 (7)<br>7-101 cd00063 (FN3)
257	157582	155633	Phage baseplate wedge subunit (T4-like gp6)	YP_009289415.1	100	73	PKO111	16-649 PHA02553 (6)
258	157875	157582	Phospholipase	YP_009288839.1	100	81	vB_KpnM_KpV477	3-96 cd14737 (PAAR_1)

								12-88 pfam05488 (PAAR_motif)<br>22-96 COG4104 (PAAR)
259	158369	157875	Phage protein	YP_009288838.1	100	65	vB_KpnM_KpV477	1-163 PHA02606 (5.1)
260	160111	158372	T4-like phage baseplate hub + tail lysozyme	YP_009288837.1	100	76	vB_KpnM_KpV477	175-340 cd00735 (bacteriophage_T4-like_lysozyme)<br>33-173 pfam06714 (Gp5_OB)<br>197-326 pfam00959 (Phage_lysozyme)<br>481-544 pfam03961 (DUF342)<br>4-579 PHA02596 (5)
261	160695	160108	Phage baseplate wedge	YP_009289411.1	100	71	PKO111	1-192 PHA02578 (53)<br>1-188 pfam11246 (Phage_gp53)
262	160747	161196	Phage head completion protein	YP_009289410.1	100	83	PKO111	1-149 PHA02552 (4)<br>55-139 pfam08722 (Tn7_Tnp_TnsA_N)
263	161196	162026	Phage DNA end protector during packaging	YP_004010009.1	100	77	CC31	116-276 PHA02577 (2)
264	162026	162634	Phage tail completion protein	YP_009289408.1	97	69	PKO111	7-182 PHA02576 (3)
265	162631	163335	Deoxynucleotide monophosphate kinase (EC 2.7.4.13)	YP_009148127.1	100	60	STML-198	6-234 PHA02575 (1)<br>212-234 COG0237 (CoaE)
266	163332	163565	hypothetical protein	YP_009190719.1	67	42	JD18	
267	163566	164021	T4-like phage	YP_009288829.1	100	82	vB_KpnM_KpV477	1-148 PHA02574 (57B)

			protein, T4 GC1584					
268	164115	164339	hypothetical protein	YP_009205836.1	100	86	vB_EcoM_VR5	
269	164405	164668	hypothetical protein	no significant similarity				
270	164734	164913	hypothetical protein	YP_009323342.1	100	58	WG01	
271	164920	165105	hypothetical protein	YP_009190315.1	100	54	PEi20	23-45 TIGR00078 (nadC)
272	165095	165472	Phage protein	YP_009190314.1	99	43	PEi20	
273	165472	165768	Phage protein	YP_803078.1	100	43	RB32	
274	166796	166933	hypothetical protein	no significant similarity				
275	167097	167279	hypothetical protein	YP_009030188.1	90	63	PS2	
276	167261	167512	hypothetical protein	no significant similarity				
277	167512	167964	T4-like phage protein, T4 GC1630	YP_009190311.1	98	89	PEi20	1-146 PHA02573 (30.3)<br>9-144 pfam08010 (Phage_30_3)<br>5-50 cd15457 (NADAR)<br>7-41 COG3236 (ybiA)
278	167961	168197	hypothetical protein	YP_009190310.1	100	54	PEi20	YP_009288814.1
279	168927	169460	Phage protein	YP_009288814.1	45	55	vB_KpnM_KpV477	
280	169549	169743	hypothetical protein	YP_009190306.1	98	52	PEi20	
281	169740	169982	hypothetical protein	YP_009190312.1	72	41	PEi20	
282	170150	170281	hypothetical protein	no significant similarity				
283	170343	170579	hypothetical protein	YP_009190303.1	100	60	PEi20	

© 2017 by the authors. Submitted for possible open access publication under the terms and conditions of the Creative Commons Attribution (CC BY) license (http://creativecommons.org/licenses/by/4.0/).
